# A GWAS on *Helicobacter pylori* strains points to genetic variants associated with gastric cancer risk

**DOI:** 10.1186/s12915-018-0550-3

**Published:** 2018-08-02

**Authors:** Elvire Berthenet, Koji Yahara, Kaisa Thorell, Ben Pascoe, Guillaume Meric, Jane M. Mikhail, Lars Engstrand, Helena Enroth, Alain Burette, Francis Megraud, Christine Varon, John C Atherton, Sinead Smith, Thomas S. Wilkinson, Matthew D. Hitchings, Daniel Falush, Samuel K. Sheppard

**Affiliations:** 1 Microbiology and Infectious Disease Group, Swansea University Medical School, Swansea University, Swansea, UK; 2 Antimicrobial Resistance Research Centre, National Institute of Infectious Diseases, Toyama, Japan; 3 Department of Microbiology, Tumour and Cell Biology, Karolinska Institutet, Stockholm, Sweden; 4 The Milner Centre for Evolution, Department of Biology and Biochemistry, University of Bath, Bath, UK; 5 School of Biosciences, College of Biomedical and Life Sciences, Cardiff University, Cardiff, CF10 3AX UK; 6 Systems Biology Research Group, School of Biosciences, University of Skövde, Skövde, Sweden; 7 Department of Gastroenterology, Centre Hospitalier Interrégional Edith Cavell/Site de la Basilique, Brussels, USA; 8 Laboratoire de Bactériologie, Centre National de Référence des Campylobacters et des Hélicobacters, Place Amélie Raba Léon, 33076 Bordeaux, France; 9 INSERM, University Bordeaux, UMR1053 Bordeaux Research In Translational Oncology, BaRITOn, 33000 Bordeaux, France; 10 Nottingham Digestive Diseases Centre and National Institute for Health Research (NIHR) Nottingham Biomedical Research Centre, Nottingham University Hospitals NHS Trust and University of Nottingham, Nottingham, UK; 11 Department of Clinical Medicine, School of Medicine, Trinity College Dublin, Dublin 2, Ireland

**Keywords:** *Helicobacter pylori*, GWAS, Gastric cancer

## Abstract

**Background:**

*Helicobacter pylori* are stomach-dwelling bacteria that are present in about 50% of the global population. Infection is asymptomatic in most cases, but it has been associated with gastritis, gastric ulcers and gastric cancer. Epidemiological evidence shows that progression to cancer depends upon the host and pathogen factors, but questions remain about why cancer phenotypes develop in a minority of infected people. Here, we use comparative genomics approaches to understand how genetic variation amongst bacterial strains influences disease progression.

**Results:**

We performed a genome-wide association study (GWAS) on 173 *H. pylori* isolates from the European population (hpEurope) with known disease aetiology, including 49 from individuals with gastric cancer. We identified SNPs and genes that differed in frequency between isolates from patients with gastric cancer and those with gastritis. The gastric cancer phenotype was associated with the presence of babA and genes in the cag pathogenicity island, one of the major virulence determinants of *H. pylori*, as well as non-synonymous variations in several less well-studied genes. We devised a simple risk score based on the risk level of associated elements present, which has the potential to identify strains that are likely to cause cancer but will require refinement and validation.

**Conclusion:**

There are a number of challenges to applying GWAS to bacterial infections, including the difficulty of obtaining matched controls, multiple strain colonization and the possibility that causative strains may not be present when disease is detected. Our results demonstrate that bacterial factors have a sufficiently strong influence on disease progression that even a small-scale GWAS can identify them. Therefore, *H. pylori* GWAS can elucidate mechanistic pathways to disease and guide clinical treatment options, including for asymptomatic carriers.

**Electronic supplementary material:**

The online version of this article (10.1186/s12915-018-0550-3) contains supplementary material, which is available to authorized users.

## Background

The bacterium *Helicobacter pylori* can colonize the stomach for years without causing any symptoms [1], but its presence is associated with several serious clinical diseases including peptic ulcer, gastric cancer and MALT lymphoma. Progression to clinical disease depends in part upon diet, environment and host factors [2, 3] as well as the genotypes of the bacteria [4].

A detailed understanding of the pathways to disease and *H. pylori*’s role at each stage has the potential to inform treatment options. For example, eradication of *H. pylori* is recommended for asymptomatic cases [5] in parts of the world where gastric cancer risk is high, but eradication can be difficult and expensive, especially due to increasing antimicrobial resistance [6]. A better understanding of the role of *H. pylori* in causing disease and identification of virulent strains would allow intervention to be targeted at patients most at risk of the subsequent disease.

Genome-wide association studies (GWAS) have become popular in human genetics as a way of investigating the basis of susceptibility to particular diseases [7]. Individuals with the disease and matched controls are genotyped, and statistical tests are performed to identify variants that are disease-associated. Functional characterization of the associated regions provides insight into how disease develops and allows the identification of “at risk” individuals for prophylactic treatments. GWAS can also be applied to bacteria [8, 9]. There are several challenges that are shared with human association studies, such as the difficulty of accurately delineating phenotypes and obtaining matched controls as well as potential false positives resulting from population structure and genetic linkage.

There are also challenges specific to *H. pylori* GWAS. For example, causative strains may be absent when disease is detected, particularly because precancerous lesions change the physiology of the stomach and can destroy the niche that the bacterium previously occupied. Furthermore, the pathway from asymptomatic carriage to disease can vary, as can the outcome. For example, antral-predominant gastritis is often associated with a higher level of acid production and is more likely to evolve into duodenal ulcer or MALT lymphoma, whereas corpus-predominant atrophic gastritis is associated with a lower level of acid production and can lead to gastric ulcer or gastric cancer [10].

Here, we assemble an *H. pylori* isolate genome collection from clinically characterized samples, including from individuals with non-atrophic gastritis, atrophic gastritis, intestinal metaplasia, and gastric cancer. We applied GWAS techniques that have been developed for other bacteria, limiting analysis to isolates from the hpEurope population to avoid confounding by population structure. We show that signals of association are sufficiently strong to identify putative cancer-associated elements using a small number of samples, highlighting the potential of bacterial GWAS to inform treatment of *H. pylori* infection.

## Results

### Population structure

**Fig. 1:**
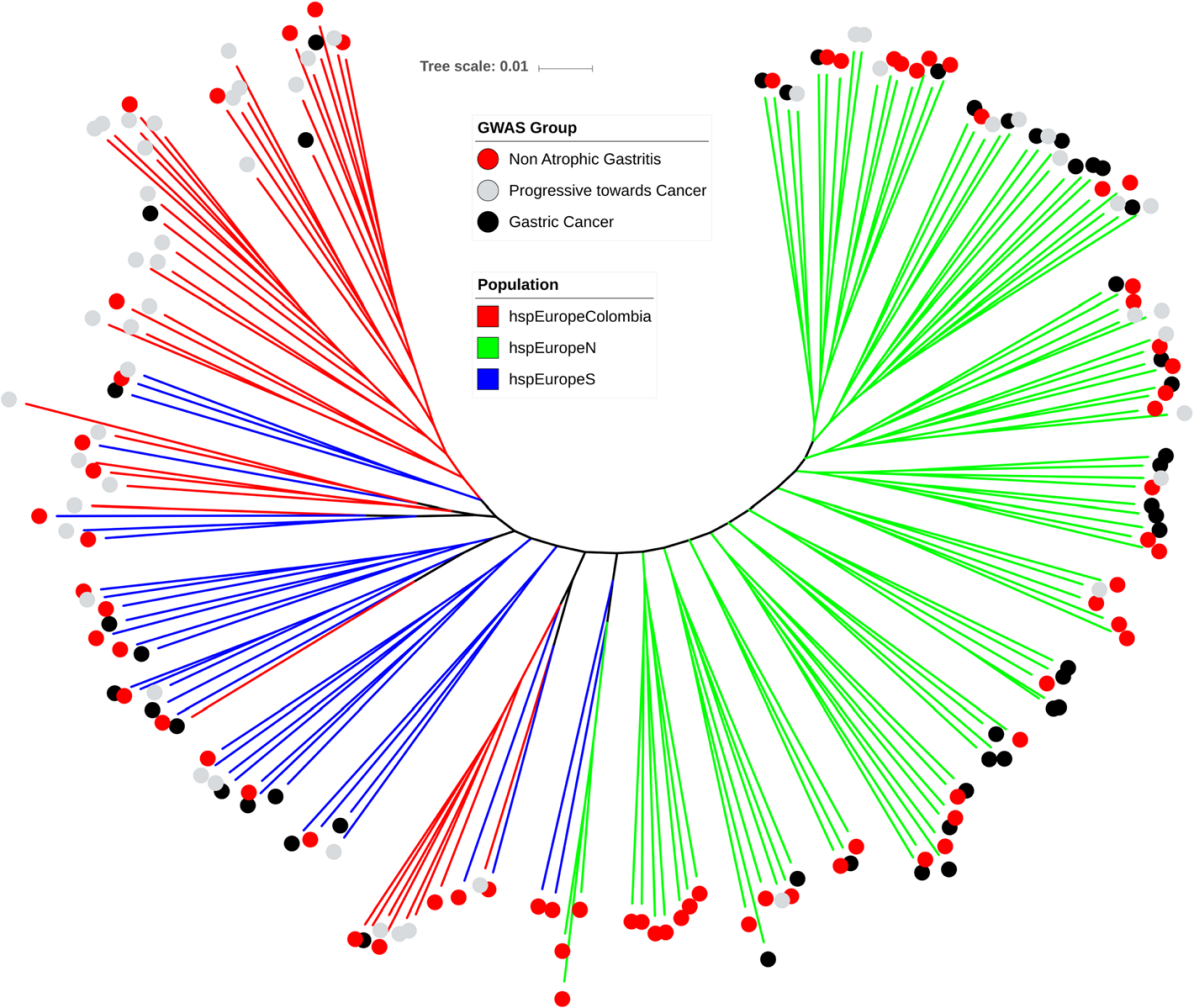
Neighbour-joining tree based on whole genome sequence alignment of all 173 strains from hpEurope-derived populations. Branches are shaded according to the population determined by fineSTRUCTURE analysis [17]. Labels reveal the patient disease background grouped into three categories: non-atrophic gastritis, progressive towards gastric cancer and gastric cancer. The scale bar represents a genetic distance of 0.02

The final dataset for analysis comprised 173 strains with clinical designations of non-atrophic gastritis, progressive to cancer and gastric cancer. These strains were obtained from a larger collection of 565 *H. pylori* genomes after excluding strains that either did not have an appropriate clinical designation or were not assigned to the hpEurope population in a fineSTRUCTURE [11] analysis (Additional file 1: Figure S1). There is substantial population structure within the 173 isolates. GC, Prog and NAG isolates were found in multiple places on the tree, and isolates from Northern Europe clustered at one end of the tree and those from Southern Europe and South America at the other (Fig. 1). The first principal component is 2.2% of the total genetic variance and basically corresponds to the difference between hspEuropeN and others. Isolates from patients with gastric cancer are distributed across the tree, and after decomposing the genetic data into principal components, none was found to be significantly associated with the cancer phenotype.

### Genome-wide association study

**Fig. 2:**
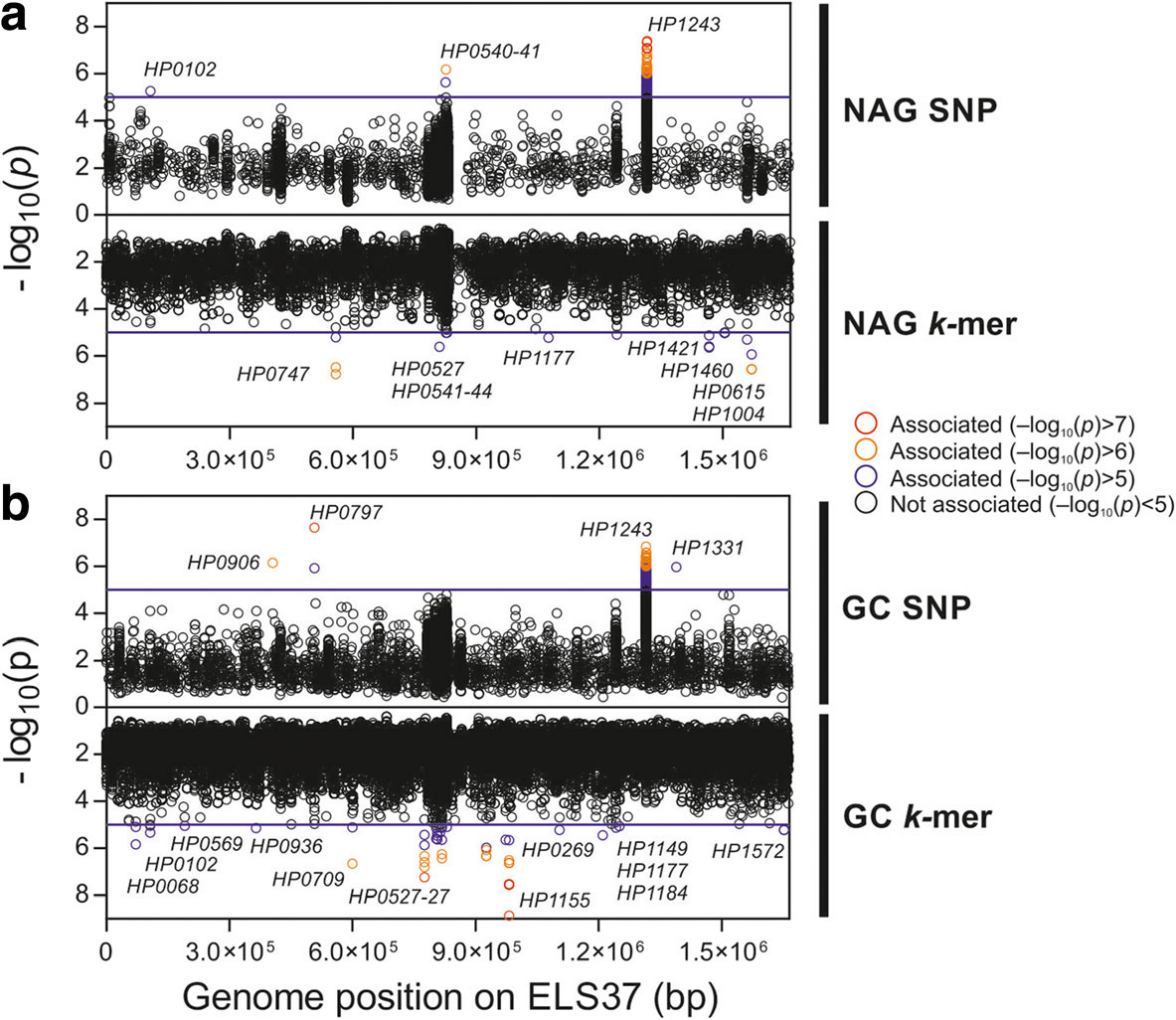
Location of genetic elements associated with gastric cancer on ELS37 genome (GCA_000255955.1). GWAS comparing isolates from patients with (**a**) non-atrophic gastritis to those with gastric cancer and precancerous progression and (**b**) gastric cancer to those with non-atrophic gastritis and precancerous progression. Two GWAS were performed with bugwas software for each panel, one based on SNPs (upper panels) and the other based on k-mers (lower panels). Positions of the genomic elements are represented on the horizontal axis expressed. Log 10 of *p* value for each hit is recorded on the vertical axis. The blue line indicates a *p* value ≤ 10^−5^

Bugwas [8] was used to identify motifs that were significantly associated with the cancer phenotype in two phenotype association comparisons: (i) GC vs Prog and NAG and (ii) NAG vs Prog and GC, and on SNP and k-mer level, resulting in four separate tests. In the first GWAS, GC vs Prog and NAG, we identified 9882 SNPs and 49,903 k-mers with a frequency difference > 20% between groups. In the second GWAS, NAG vs Prog and GC, there were 9273 SNPs and 26,581 k-mers with a frequency difference > 20%. GWAS hits were filtered by *p* value, resulting in a total of 642 hits (432 SNPs and 210 k-mers) with a frequency difference > 20% and a *p* value ≤ 10^−5^ (Fig. 2, Table 1). A large number of hits are found in a single gene, so these 642 hits are spread in only 32 genes. Of these, 6 genes recorded hits in two of the four GWAS tests: *HP0102*, *HP0468*, *cag11* (*HP0531*), *cag12* (*HP0532*), *cag20* (*HP0541*), *cagE*/*cag23* (*HP0544*), *hopQ* (*HP1177*) and *babA* (*HP1243*).

**Table 1:** Summary of the hits obtained in the genome-wide association studies based on 173 strains from hpEurope-derived sub-populations based upon patient disease phenotype

	Number of hits with *p* value		Number of genes with hits of *p* value	
GWAS experiment	≤ 10^−5^	≤ 10^− 6^	≤ 10^− 5^	≤ 10^− 6^
Gastric cancer vs others (k-mer)	166	39	20	6
Non-atrophic gastritis vs others (k-mer)	44	15	10	2
Gastric cancer vs others (SNP)	237	33	4	3
Non-atrophic gastritis vs others (SNP)	195	31	4	2

Amongst the 32 genes with hits at a *p* value ≤ 10^−5^ (Additional file 5: Table S3), 13 were in genes with putative functions associated with virulence of *H. pylori* such as CagPAI and type IV secretion system [12, 13] (11 genes), buffering of gastric acid [14] (*ureG*) or adherence [15] (*babA*). Further, 8 genes had putative functions that may also be indirectly linked to virulence, such as colonization (*hpaA*), motility (*fliK*) or more generally membrane and outer membrane proteins (5 genes). A total of 12 genes were either hypothetical proteins with unknown functions (2 genes), or had functions not previously linked to virulence; amongst them were genes associated with enzymes (6 genes), ribosome maturation factors (2 genes), transporters (1 gene) and a DNA-binding protein (1 gene).

**Fig. 3:**
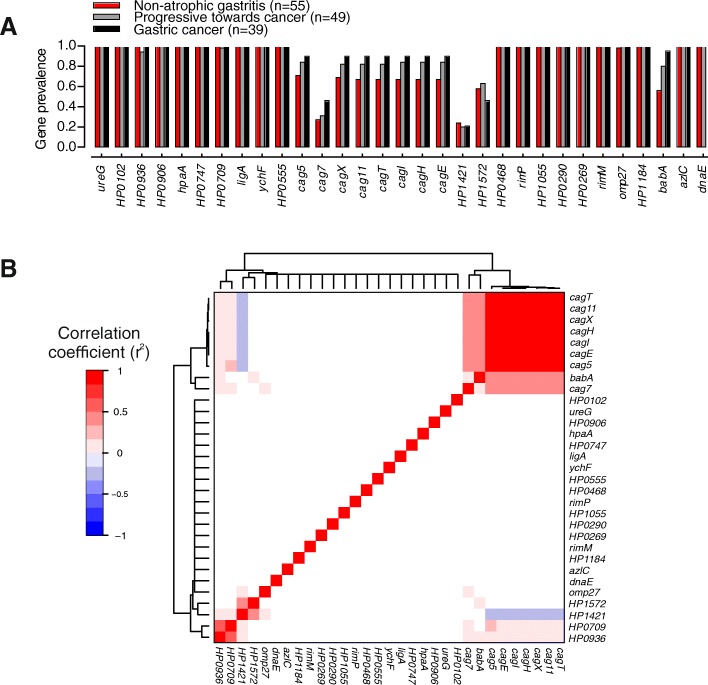
Prevalence of genes highlighted by GWAS in *H. pylori* genomes. **a** Prevalence of genes containing a GWAS hit with *p* value < 10^−5^ in three groups of isolates: non-atrophic gastritis isolates (red, *n* = 55 genomes), progressive toward cancer isolates (grey, *n* = 49) and gastric cancer isolates (black, *n* = 39), and defined as the ratio of number of isolates in each group harbouring the gene and the total number of isolates in each group. **b** Matrix of correlation of pairs of gene prevalence patterns in 143 *H. pylori* genomes. Red indicates that two genes have a high positive correlation of their patterns of presence and absence in all genomes examined and blue indicates a negative correlation. White indicates core genes that did not vary in prevalence in the dataset and for which correlations could not be calculated

Multiple cancer risk-associated k-mers or SNPs can be present in a single gene as the GWAS approach targets variation in the frequency of DNA sequence motifs within the population rather than the whole genes themselves. This is particularly apparent for accessory genes (present or absent) such as those within CagPAI for example, as all the elements that map to these genes will be either present or absent together. However, not all elements (SNPs or k-mers) will necessarily have the same association significance. This is because the *p* value is dependent upon the degree to which the genetic element segregates by the phenotype under study, compared to expectation based on the clonal frame of the population. Therefore, other sequence variation that does not meet these criteria will not have a low *p* value. The prevalence and co-occurrence of genes containing a GWAS hit with *p* value < 10^−5^ was investigated in NAG, Prog and GC isolates (Fig. 3). As expected, CagPIA genes were commonly found together when present and were also positively correlated with the presence of the *babA* gene.

The most significantly associated 118 GWAS hits were in 12 genes (64 SNPs and 46 k-mers) and had a frequency difference > 20% and a *p* value ≤ 10^−6^ (Table 1). Only one gene, *babA*, was a hit with a *p* value ≤ 10^−6^ in two GWAS experiments (SNP GC vs rest and SNP NAG vs rest). In order to keep the number of genes for risk score calculation low, only these 12 genes were investigated further.

### Risk genotypes

Sequence variation amongst the 12 bacterial genes containing k-mers or SNPs with the most significant association (*p* value ≤ 1 × 10^−6^, Fig. 2) was further investigated amongst isolates associated with different disease outcomes. For one of these genes, *HP0555*, specific nucleotides were enriched amongst the Prog isolates compared to both NAG and GC isolates. This highlights the potential that different nucleotide variations may be important at different stages in the complex disease progression but may occur by chance. For two of the other genes with k-mer hits, *HP1004* and *HP0906*, distinct coding sequences from ELS37 aligned against a single gene resulting in false positive hits observed in *HP1004* and *HP0906*. The remaining 9 genes revealed a total of 11 risk genotypes that were highly enriched among gastric cancer strains (Table 2). Amongst them, 4 corresponded to accessory elements that were more commonly present in isolates from patients with gastric cancer. Hits in these genes were spread across the whole genes (Additional file 2: Figure S2). The remaining 7 risk genotypes corresponded to variation in homologous sequence. Hits in these genes were limited to small areas of the genes, and strong hits were surrounded by weaker hits (Additional file 3: Figure S3). The ratio of synonymous to non-synonymous SNPs (dN/dS) was calculated for these genes and compared to the dN/dS for 7 multilocus sequence typing (MLST) genes not thought to be under strong diversifying selection. This showed evidence of significant enrichment (*p* value ≤ 0.03) for non-synonymous SNPs amongst cancer-associated sequence variation (dN/dS = 0.588) compared to MLST genes (dN/dS = 0.364). Ratio for randomly selected genes in the core genome was consistent with the MLST genes (data not shown). Regardless of this, not all of the cancer-associated SNPs represented non-synonymous variation in homologous sequence (4 of 7).

**Table 2:** Cancer risk genotypes identified in genome-wide association studies of 173 hpEurope isolates

Gene name^1^	*p* value (min)	Risk genotype	Position^2^	Safe genotype	Frequency^3^	Effect on amino acid sequence^4^	Function
*HP1055* [981621–982,565] (−)	1.4.10^−9^	A	798	C	0.469/0.125	S, associated with G to A substitution at position 797: non-synonymous with T in safe, A in risk	Outer membrane protein
*HP0797* [506543–507,325] (+)	2.24.10^−8^	C + T	325 and 334	T + G	0.592/0.181	NS: L/S in safe, F/A in risk	Neuraminyllactose-binding hemagglutinin (HpaA) [29]
*HP1243,babA1* [1314192–1,316,405] (−)	3.99.10^−8^	Presence	All genes	Absence	0.94/0.51		BabA (outer membrane protein) [16]
*HP0747* [317158–317,757] (+)	1.69.10^− 7^	GGAA	934 to 937	AAAA/GGAG	0.531/0.264	NS: KA in safe, GT in risk	tRNA (guanine-N(7)-)-methyltransferase
*HP0709* [598549–599,451] (−)	2.13.10^− 7^	A	145	G	0.327/0.153	NS: D in safe, N in risk	Adenosyl-chloride synthase
		A	159	G	0.959/0.792	S	
*HP0532,cag12* [817677–818,519] (+)	3.62.10^− 7^	Presence	All genes	Absence	0.92/0.61		CagT protein (Censini, 1996)
*HP0468* [925539–927,026] (+)	4.59.10^− 7^	CGCC	705 to 708	CACG/TGCG	0.694/0.514	NS: T in safe, A in risk	Unknown
		A	729	G	0.796/0.5	S	
*HP0531,cag11* [816985–817,641] (+)	5.4.10^−7^	Presence	All genes	Absence	0.92/0.61		CagU protein (Censini, 1996)
*HP0541,cag20* [825334–826,446] (−)	6.6.10^−7^	Presence	All genes	Absence	0.92/0.61		CagH protein (Censini, 1996)

Risk and safe genotypes are overrepresented amongst isolates from patients with gastric cancer and non-atrophic gastritis respectively, with *p* value corresponding to the minimum in each gene (*p* value ≤ 1 × 10^−6^)

^1^Position in ELS37 genome [ ], + and – strand is denoted in ( )

^2^Position in gene

^3^Frequency GC strains/NAG strains

^4^The effect on the amino acid sequence is indicated as synonymous (S) and non-synonymous (NS)

**Fig. 4:**
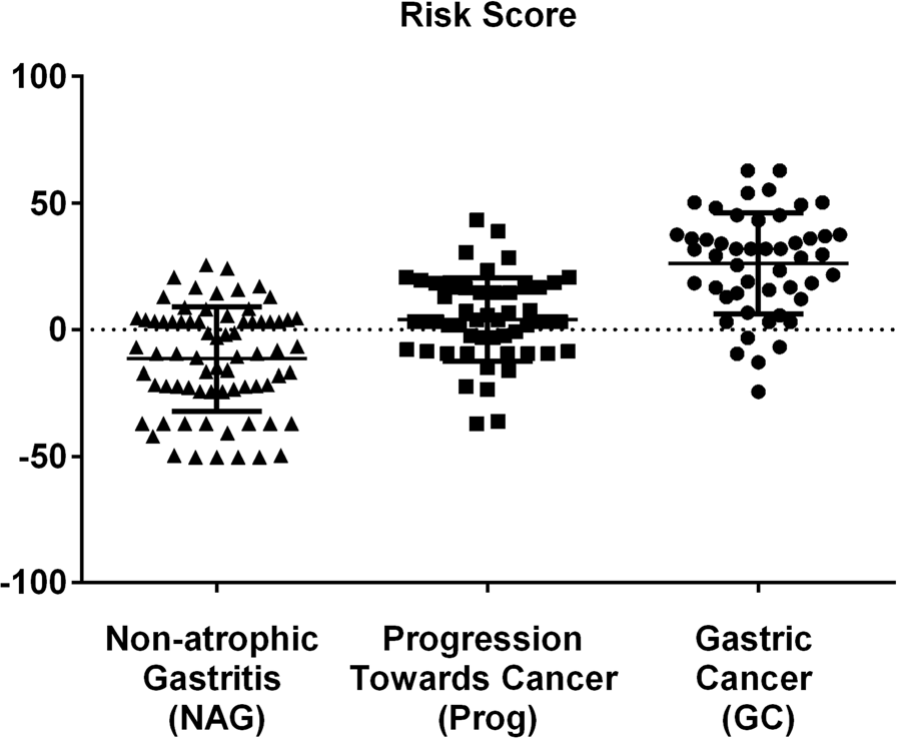
Repartition of risk scores on 173 strains from hpEurope-derived sub-populations, according to patient disease background. Each point corresponds to the risk score associated with a single strain. This risk score was calculated based on the presence of risk or safe genotype for each of the 9 genes considered (Table 2)

Three of the associated cancer risk genotypes in the CagPAI genes (*cag11*, *cag12* and *cag20*) were correlated and therefore not independent, based upon Pearson’s correlation (Fig. 3). To limit the weight of these correlated genes, an average of the 3 was used in the calculation of the risk score. As expected, the distribution of risk scores in our dataset was significantly associated with disease progression (ANOVA, *p* value < 0.0001) (Fig. 4). Specifically, patients from which *H. pylori* isolates presented a risk score below − 25 may be unlikely to develop gastric cancer, as no isolate from such patients had a risk score below − 24.37. Seventeen patients have a risk score under this limit and on this basis might be considered lower priority for *H. pylori* eradication, depending on other factors. However, it should be emphasized that we have calculated the risk score for the same isolates used to perform the GWAS rather than with an independent validation panel. Therefore, while our results highlight the potential utility of risk scores in evaluating treatment options, our current implementation should not be used in clinical management.

## Discussion

It has been known for some time that the presence of certain genes in *H. pylori* strains increases the risk that the host will develop gastric cancer [16], and for genes such as those in the Cag pathogenicity island, the mechanism is well-characterized [13]. Technical advances in high-throughput DNA sequencing and the increasing availability of whole genome data for diverse *H. pylori* isolate collections provide opportunities for quantitative genomic analysis of population structure [17] and the genetic determinants of important disease phenotypes.

Host and environmental factors and different pathways to disease impose additional complexity when identifying cancer-associated genes in *H. pylori*, compared to standard binary bacterial GWAS [9]. However, even in the relatively small isolate collection in this study, variation in known cancer-associated genes, including CagPAI, was identified, as well as in genes that have not previously been associated with virulence.

Cancer-associated nucleotide variation was largely the result of the presence of accessory genes and enrichment for non-synonymous SNPs in homologous sequence. While interpretation of sequence or whole gene insertion and variation that causes changes in protein sequences is easier to interpret in relation to functional variation, 3 of the 12 most significant GWAS hits were synonymous SNPs associated with gastric cancer isolates. There are several potential explanations for these hits. First, synonymous sequence variation associated with isolates from gastric cancer patients can be in linkage disequilibrium with non-synonymous SNPs, which may give lower *p* values despite being the functional drivers of the association. Second, synonymous mutations can have functional effects [18], and there is evidence of selection acting across the *H. pylori* genome [19]. Third, frameshifts or uncharacterized start codons lead to misinterpretation of non-synonymous SNPs as synonymous. Finally, some may represent false positives.

Investigating the putative function of genes containing sequence elements associated with cancer can provide clues about the bacterial phenotypes that promote the development of disease in infected individuals, as well as providing novel targets for diagnosis and intervention. As expected from previous studies [16], our GWAS identified elements in CagPAI genes (*cag11*, *cag12* and *cag20*) and *babA* that were associated with isolates from patients with gastric cancer. CagPAI-positive strains are known to predominate in gastric cancer patients [13] and are associated with enhanced immune response through diverse pathways starting with the injection of CagA through a type IV secretion system into host epithelial cells [20].

The blood group antigen-binding adhesin BabA is an outer membrane protein linked to the activity of the CagPAI island through adhesion to the host cells [21]. The binding characteristics of babA in different strains are known to vary in relation to the blood types in host populations [22], showing an important and specific evolutionary pressure on *H. pylori* isolates [23]. BabA expression is regulated by phase variation and recombination between *babA* and highly homologous genes *babB* and *babC* with important consequences for binding characteristics and affinities [22]. The homology between *bab* genes, which can all be absent or present as duplicates, imposes challenges for the de novo assemblies of Illumina short reads in this study. Specifically, of 173 sequences annotated, 48 contained full *babA* sequence, while for other genomes, only partial *bab* gene sequence(s) were annotated, often at the end of a contig, reflecting challenges associated with genome assembly and the interchangeability of these loci.

In addition to quantifying the effect of known *H. pylori* virulence genes, the GWAS approach employed here also provided evidence for a role for genes that have not previously been linked to gastric cancer. In addition to BabA, a second outer membrane protein, encoded by *HP1055*, was strongly associated with cancer. While little is known about the specific function of this gene, other than its essentiality demonstrated in transposon mutagenesis experiments [24], outer membrane proteins can influence host-bacteria interactions mediating virulence by modulating colonization and adherence to the host cells and facilitating secretion of virulence factors. A possible link to enhanced cancer risk is that *HP1055* contains sequence enriched for African ancestry [17] and conflicts between the host and bacterial genetic population are a risk factor for gastric cancer [25, 26].

The function of the gene harbouring the second strongest cancer-associated GWAS hit in this study, *HpaA* (HP0797), is the subject of some debate. Originally described as a sialic acid-binding protein involved in adhesion [27], it is now thought to have a role as a lipoprotein [28] and is essential for stomach colonization in an in vivo mouse model [29]. A speculative role in disease progression could be related to the strong immunogenic properties of the HpaA protein [30], and the substitutions described in our study alter the orientation of one of the helix formations (Additional file 4: Figure S4) which may be related to changes in protein function. This protein is considered as a target for vaccine development [31, 32].

Other *H. pylori* genes, in which a highly significant association was found with gastric cancer included *trmB (HP0747)*, and the less well-annotated *HP0709* and *HP0468*. *trmB*, homologous to *E. coli Yggh*, encodes a predicted S-adenosylmethionine-dependent methyltransferase regulated by the *H. pylori* orphan response regulator *HP1021* [33], presumably involved in the regulation of acetone metabolism. It has also been identified as a gene with overrepresented radical substitutions in fast-evolving regions [34]. *HP0709* encodes an enzyme that is involved in either methylation of DNA and proteins or in the synthesis of branched amino acids valine, leucine and isoleucine. However, the exact function is not certain and conflicting annotations [35] make protein structure prediction problematic, making it difficult to compare alleles in our dataset beyond the identification of cancer-associated SNPs. *HP0468* encodes a hypothetical protein, poorly conserved outside the *Helicobacter* genus. It is upregulated by molecular hydrogen in chemolithoautotrophically enhanced growth of *H. pylori*, but its exact function is yet to be determined [36].

The GWAS approach used in this study supports known genotype-phenotype associations as well as providing information about specific genetic variations and highlighting a potential role for candidate genes that have not previously been related to gastric cancer. Quantitative GWAS using natural *H. pylori* populations is complicated by numerous host and pathogen changes in the progression from asymptomatic carriage to gastric cancer. This involves changes to stomach cells, pH, the extracellular mucus layer and changes in the selective landscape for the pathogen, promoting strains with functions related to adherence, motility and immune evasion that can survive in the harsh changing acidic environment. These changes make the phenotype complex, especially since the strains that are most responsible for disease progression need not be those that are isolated from gastric cancer patients. Nevertheless, our results are encouraging since they suggest that the most important factors may have large effect on progression and therefore be detectable in GWAS cohorts despite inevitable imperfections in the sampling design due to the difficulty of finding well-matched cases and controls.

## Conclusions

In addition to providing information on the biology of disease progression, GWAS may be of direct relevance in the clinic. By sequencing the strains before eradicating them, we could assess the risk of gastric cancer, enabling closer surveillance of those with increased risk while avoiding unnecessary treatment for the others, therefore reducing the proportion of highly pathogenic strains in the overall *H. pylori* population and mitigate the spread of antimicrobial resistance.

## Methods

### Isolates and genome sequencing

A total of 565 *H. pylori* isolate genomes were analysed in this study (Additional file 5: Table S1). This dataset comprised 122 strains isolated from clinical samples including strains isolated in France (from patients from different areas of France enrolled in studies carried out by the GEFH, the GELD and FFCG, and the GELA), Belgium (from patients attending the endoscopy clinic of CHIREC—sites de la Basilique and E. Cavell, Brussels), the UK (biopsies from patients attending for upper GI endoscopy at Nottingham University Hospitals NHS Trust), Sweden (eight hospitals) and Dublin (the Meath Foundation Research Laboratory, Tallaght Hospital, Dublin), and 444 publically available genomes from published papers [17] and the NCBI database. Swedish isolates were a subset of the collection assembled by Enroth and colleagues in a previously published study [37]. For isolates sequenced for this study, bacteria were sampled from patients presenting with gastric cancer, gastritis, gastrointestinal stromal tumour (GIST) or no symptoms, from 1995 to present by gastric biopsy and grown on *H. pylori*-selective medium (Dent plates) at 37 °C in a microaerophilic environment (CampyGen or microaerophilic cabinet) for 5 to 10 days. Isolates from gastric MALT lymphoma or other non-adenocarcinoma forms of cancer (apart from 1 GIST isolate) were excluded from analysis. Colonies were isolated as single colonies and subcultured on fresh blood agar plates to obtain sufficient growth, and for genomic DNA extraction, DNA was quantified using a NanoDrop spectrophotometer, as well as the Quant-iT DNA Assay Kit (Life Technologies, Paisley, UK) before sequencing. High-throughput genome sequencing was performed using a HiSeq 2500 machine (Illumina, San Diego, CA, USA), and the 100-bp short read paired-end data was assembled using the de novo assembly algorithm, Velvet [38] (version 1.2.08). The VelvetOptimiser script (version 2.2.4) was run for all odd k-mer values from 21 to 99. The minimum output contig size was set to 200 bp with default settings, and the scaffolding option was disabled. The average number of contiguous sequences (contigs) for genomes sequenced in this study was 111 with an average total assembled genome size of 1,630,194 bp and an average N50 length of 55.98 kbp. Short reads for the 107 genomes sequenced and assembled in Swansea are available from the NCBI short read archive (SRA) associated with BioProject: PRJNA395900. All 565 contiguous assemblies of whole genome sequences were individually archived on the web-based database platform BIGSdb [39] and are available at the public data repository figshare (https://figshare.com/articles/Helicobacter_pylori_from_clinical_gastric_infection/5245837).

### Comparative genomics

Individual genes from the 26,695 *H. pylori* reference genome were locally aligned to the 776 *Helicobacter pylori* genomes available at the time of analysis using default BLAST parameters implemented in BIGSdb. A gene was recorded as present when the local alignment had at least 70% sequence identity on at least 50% of the sequence length. This allowed gene discovery, sequence export and local gene-by-gene alignments using MAFFT [40], as previously described [41, 42]. Sixty strains that were not from a human clinical source and 5 strains with a number of genes below 1000 were removed and a tree was constructed from an alignment of the remaining strains using FastTree v2.0 [43]. One hundred forty-six clones were removed from the analysis based on the clustering observed on the tree. The remaining 565 strains constituted our working dataset, and the population structure amongst these strains was inferred from genome-wide haplotype data using chromosome painting and fineSTRUCTURE [11], as in previously published *H*. *pylori* genome analysis [44]. Briefly, donor and recipient DNA chunks were inferred for each recipient haplotype using ChromoPainter (version 0.04). The number of recombination-derived chunks from each donor to each recipient was summarized in a co-ancestry matrix. fineSTRUCTURE (version 0.02) was run with 100,000 iteration burn-in and 100,000 MCMC iterations to cluster isolates based on the co-ancestry matrix. Principal component analysis was carried out on our data using the standard PCA implemented in Eigensoft. Specifically, on all biallelic data after pruning of SNPs with *r*^2^ > 0.7, Popstats (“GitHub - pontussk/popstats: Population genetic summary statistics,” n.d.) were used to calculate D-statistics and specify previously described *H. pylori* populations [17].

Isolate genomes were partitioned into groups based upon metadata from patient information collected as part of this study or taken from existing publications. To be able to identify risk factors of the carcinogenic progression, three groups were applied: (i) isolates from patients with gastric cancer (GC), (ii) isolates from individuals with intestinal metaplasia or atrophic gastritis, which we termed “progressive to cancer” (Prog) and (iii) isolates from individuals with non-atrophic gastritis (NAG). To reduce the impact of the phylogeographic structure [17] on identification of disease-associated genetic elements, the remaining analyses focussed on the largest dataset for which patient data and geographic origin were available within one unique fineSTRUCTURE population. This included 173 hpEurope isolates (Additional file 5: Table S2). Subpopulations included in hpEurope were based on previous study and included hspEuropeColombia, hspEuropeN and hspEuropeS [17]. A phylogeny for 173 isolates was constructed for visualization of the population using the simple and efficient tree building software FastTree v2.0 [43] and annotated using iTOL v3ic [45] (Fig. 1). Input data included 1573 concatenated genes, identified in the 26,695 reference strains, aligned for all isolate genomes.

### Genome-wide association studies

The genome-wide association study (GWAS) was conducted with a pipeline based on the *bugwas* package [8], as in a recent study [46]. Briefly, in this k-mer-based approach [9], the genome sequence of each isolate was fragmented into unique, overlapping, 31-bp DNA motifs or k-mers. This allowed the identification of nucleotide variation including single nucleotide polymorphisms (SNPs), indels and the presence or absence of a whole gene or gene region associated with different phenotype groups. DNA motifs significantly associated with gastric cancer were explored after accounting for the inter-dependence of the strains and population structure. An *n* × *n* relatedness matrix summarized all genetic covariance amongst the isolate genomes, employing statistical tests for each k-mer by the linear mixed regression model, which uses the relatedness matrix to model the background random effect. Unlike related methods [9, 47], this method does not depend on a single clonal tree that is impossible to construct reliably because of the high rate of recombination in *H. pylori*. A second GWAS, also implemented in the *bugwas* package [8], was carried out based upon SNPs rather than k-mers. Only the SNPs contained in coding sequences were considered. The k-mer and SNP GWAS approaches were applied to bacterial datasets in two binary phenotype association experiments: (i) GC vs Prog and NAG isolates and (ii) NAG vs GC and Prog isolates. This gave a total of four GWAS experiments.

### Analysis of associated elements

The odds ratio and *p* value was calculated for associated elements in the GWAS experiments and the position of hits in a reference genome. Specifically, a reference pan genome was produced using Roary software [48] with default parameters, and annotation was carried out using Prokka [49]. GWAS hits, representing both core and accessory nucleotide variation, were then analysed individually to investigate the putative function of the associated genes and the effect of the variations identified in the amino acid sequence. Positions of hits in all analyses were considered using the reference strain ELS37 (GCA_000255955.1). This reference strain was chosen as being part of the GC isolates used in our study with a closed genome sequence.

A limitation of k-mer-based GWAS approaches is that they reveal significantly associated sequence within genes and not the entire gene presence and absence. For this reason, the prevalence of genes (presence/absence) containing at least one significant k-mer (*p* value ≤ 10^− 5^) was determined for genomes in our dataset (*n* = 143) using BLAST. A gene was considered present when the sequence from the genome shared more than 70% sequence homology with the corresponding gene sequence from *H. pylori* reference strain ELS37. We examined the correlation of prevalence patterns across our dataset for these genes, by using the rcorr function in the *Hmisc* R package to compute correlation coefficients and the *p* value of the correlation for all possible pairs of gene presence/absence patterns. The input was a binary matrix of presence/absence of the genes in 143 genomes.

All the genes that contained a GWAS hit at *p* value < 1 × 10^−6^ were individually investigated using BioEdit [50], based on a global alignment obtained from the GWAS. Synonymous and non-synonymous variation was identified by comparison to amino acid sequence alignments, and non-synonymous hits were further studied using figures showing repartition of amino acids in each position according to the GWAS group of each strain, using WebLogo [51]. Genes identified showed there was clear enrichment for particular alleles in GC strains. Genes with GWAS hits (*p* value < 1 × 10^−6^) were mapped to the corresponding genome position on the reference genome ELS37 (GCA_000255955.1) using Circos V0.69 [52], and the context of individual genes was characterized with BioCyc [53].

### Prediction of protein structure

For the genes where the risk alleles were associated with non-synonymous changes to the amino acid sequence of the encoded proteins, we tried to predict what impact these changes would impose on the tertiary structure of the proteins. For this purpose, we used hhpred [54] as it is implemented in the MPI Bioinformatics Toolkit [55] as of 4 June 2017 to identify the most suitable structure to model from. This structure was then used to model both the safe and risk sequence using default parameters, and the models were annotated and visualized in Swiss-PDB viewer [56].

### Risk score

The most significant GWAS hits (*p* value < 1 × 10^−6^) were used to calculate a rudimentary risk score. First, the correlation between the presence of a risk or non-risk genotype and the presence of another risk or non-risk genotype was verified for each isolate pair, using a test based on Pearson’s correlation. This correlation was used to balance the weight associated with genotypes that were not independent, such as CagPAI genes that are co-located on the genome and in strong linkage disequilibrium. A Pearson’s correlation of more than 0.9 with a *p* value < 0.05 was used to define correlated genes. For each genotype, a genotype score (*g*_s_) was determined using the following parameters. For accessory genes, 1 if the gene is present, − 1 if the gene is absent. For a nucleotide change, 1 if the risk genotype is present, − 1 if the safe genotype is present, 0 if neither is present. Then, the risk score was determined using this formula, with an average of the sum for the three genes correlated:

$$ \mathrm{Risk}\ \mathrm{score}=\sum \limits_{\mathrm{for}\ \mathrm{each}\ \mathrm{genotype}}{g}_s\times -\log \left(p\ \mathrm{value}\right) $$

## Additional files

Additional file 1: **Figure S1. **Co-ancestry matrix with population structure of 565 global *H. pylori* isolates. The colour of each cell of the matrix indicates the expected number of DNA chunks imported from a donor genome (column) to a recipient genome (row). The boundaries between named populations are marked with dotted lines. The colour ranges from low (yellow) to a large amount of DNA from the donor strain (red). Diagonal clusters with more red squares indicate chunks of DNA that are shared between the pairs of isolates. (PDF 193 kb)

Additional file 2: **Figure S2.** Distribution of the GWAS hits in the 4 accessory genes used in calculation of a risk score. All the hits with a *p* value < 0.05 are represented in the figure. Positions are based on the ELS37 genome (GCA_000255955.1). (PDF 93 kb)

Additional file 3: **Figure S3.** Representation of the GWAS hits in the 5 non-accessory genes used in calculation of a risk score. All the hits with a *p* value < 0.05 are represented in the figure. The direction of the arrow representing the gene indicates on which strand the gene was found in ELS37 genome, and the length of each arrow is proportional to the length of the ELS37 version of the gene. Hits are positioned on the genes according to their position in ELS37 version of the genes. In each gene, the top half represents hits in the GC vs rest GWAS, and the bottom half represents hits in the NAG vs rest GWAS. K-mer hits are represented as lines, and SNP hits are represented as dots. Zoomed areas correspond to the areas where the genomic variations used in the risk score were found. (PDF 1523 kb)

Additional file 4: **Figure S4.** 3D renderings of the safe and risk allele of HpaA. The 3D structure of 26,695 amino acid sequence of HpaA (*HP0797*) containing the safe allele (A) and the risk allele (B) respectively was modelled using 2I9I as template. Note that the helix formations in the area changes due to the mutations. Safe allele on the right with Leu 109 and Ser 112 (first 36 aa not included in model) and risk allele to the left with Phe 109 and Ala 112. (PDF 103 kb)

Additional file 5: **Table S1.** Isolate details for the global 565 strains dataset. Summary of geographic provenance, fineSTRUCTURE population and source for the global dataset of 565 strains. **Table S2.** Isolate details for the hpEurope GWAS dataset. Summary of metadata for the 173 strains used in the GWAS study. Host pathology, GWAS group, isolation country, isolation city or region and *H. pylori* population are given when available. **Table S3.** List of the 32 genes highlighted in at least one of the GWAS experiments. The minimum *p* value and an annotation (obtained by Prokka) is mentioned for each gene. (DOCX 89 kb)
