# Supplementary figures and images for: A GWAS on Helicobacter pylori strains points to genetic variants associated with gastric cancer risk
Source: BMC Biol. 2018 Aug 2;16:84. doi: 10.1186/s12915-018-0550-3 (PMC6090961; doi:10.1186/s12915-018-0550-3)

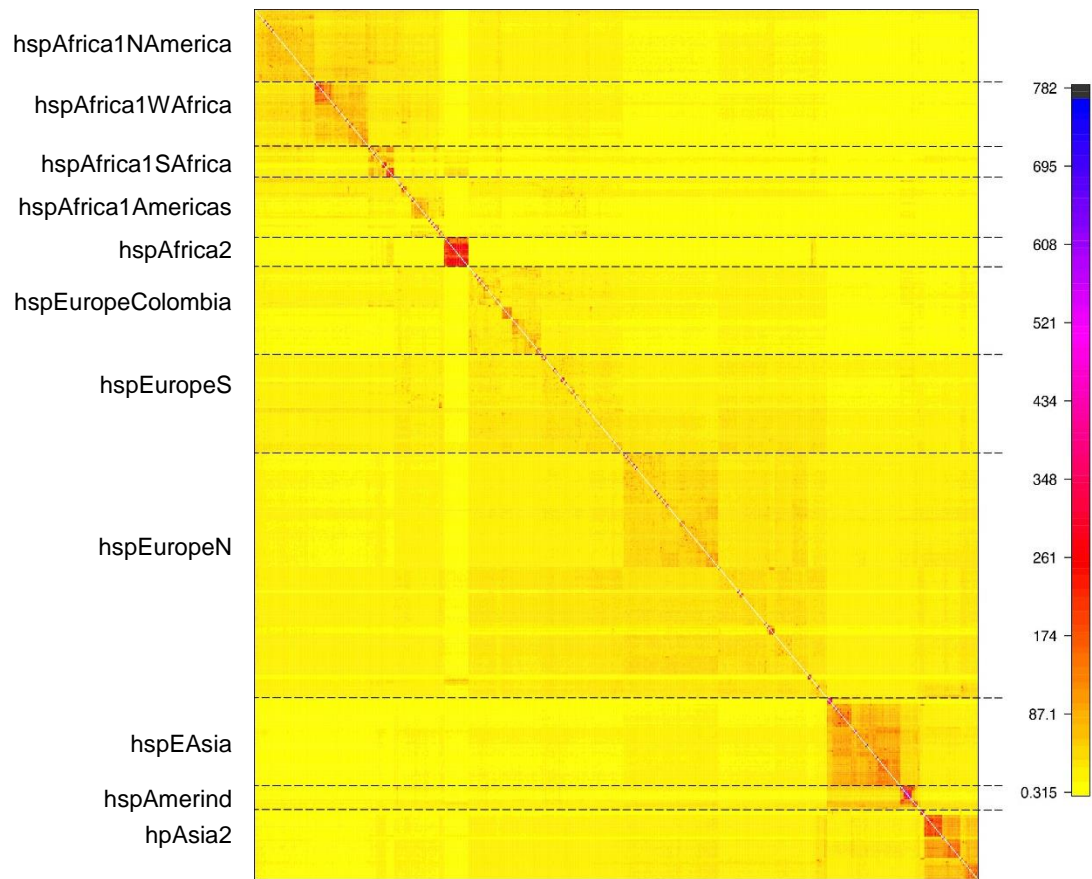

Supplement: Supplementary file 1 — Figure S1. Co-ancestry matrix with population structure of 565 global H. pylori isolates. The colour of each cell of the matrix indicates the expected number of DNA chunks imported from a donor genome (column) to a recipient genome (row). The boundaries between named populations are marked with dotted lines. The colour ranges from low (yellow) to a large amount of DNA from the donor strain (red). Diagonal clusters with more red squares indicate chunks of DNA that are shared between the pairs of isolates. (PDF 193 kb) [file 12915_2018_550_MOESM1_ESM.pdf]

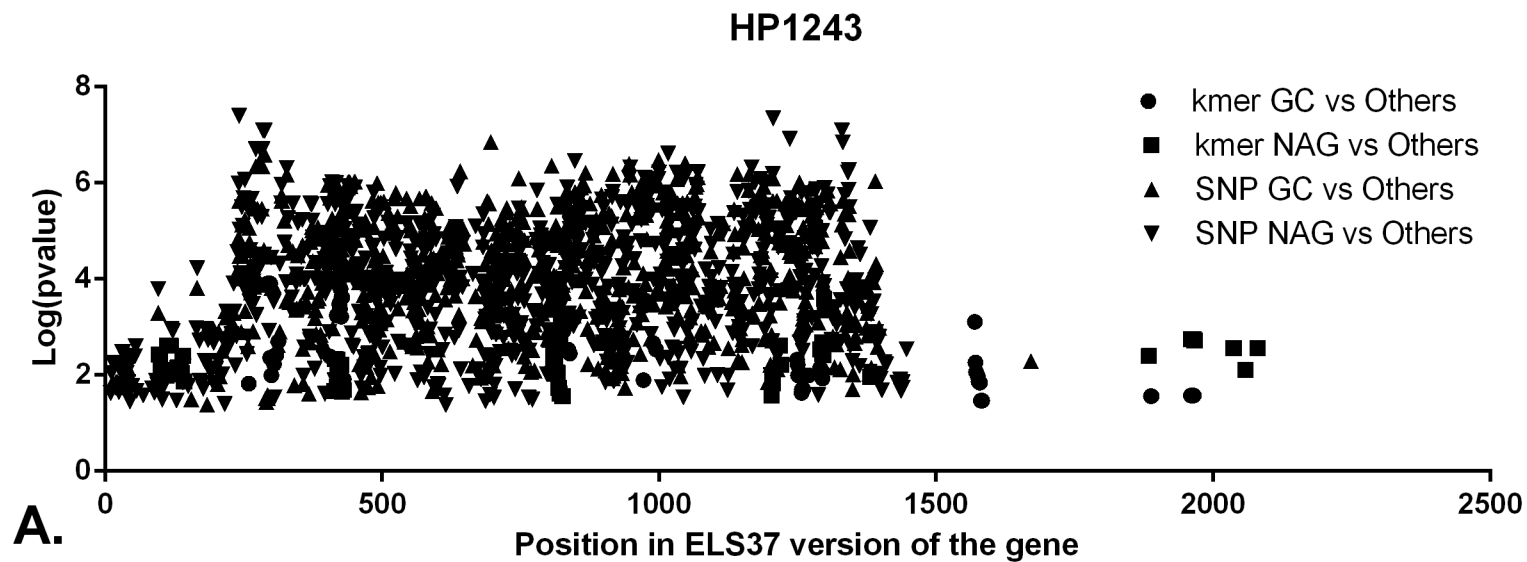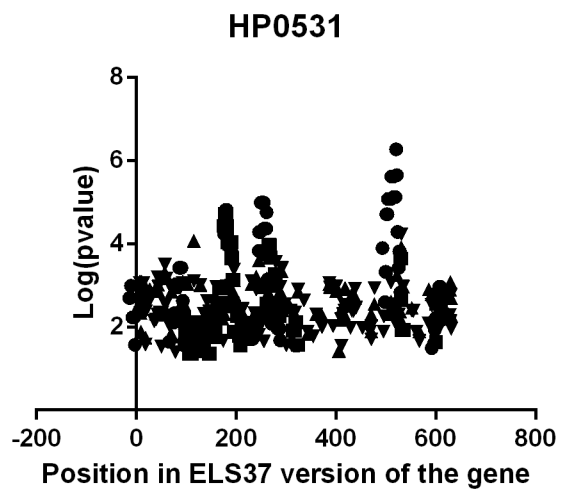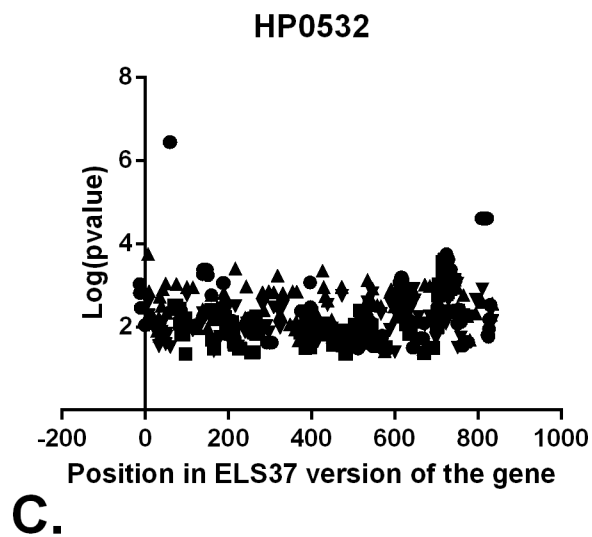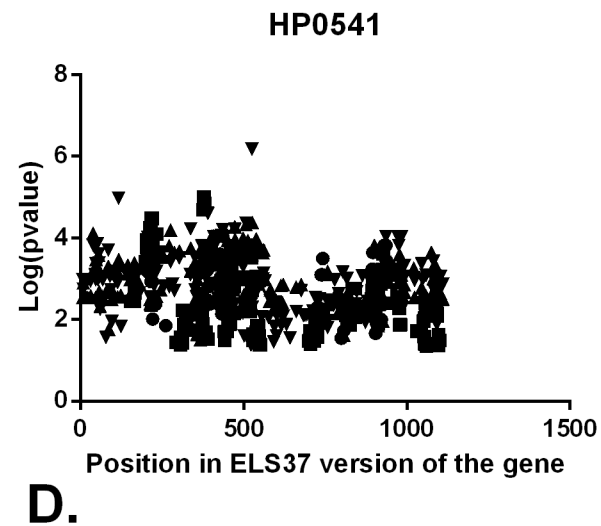

Supplement: Supplementary file 2 — Figure S2. Distribution of the GWAS hits in the 4 accessory genes used in calculation of a risk score. All the hits with a p value < 0.05 are represented in the figure. Positions are based on the ELS37 genome (GCA_000255955.1). (PDF 93 kb) [file 12915_2018_550_MOESM2_ESM.pdf]

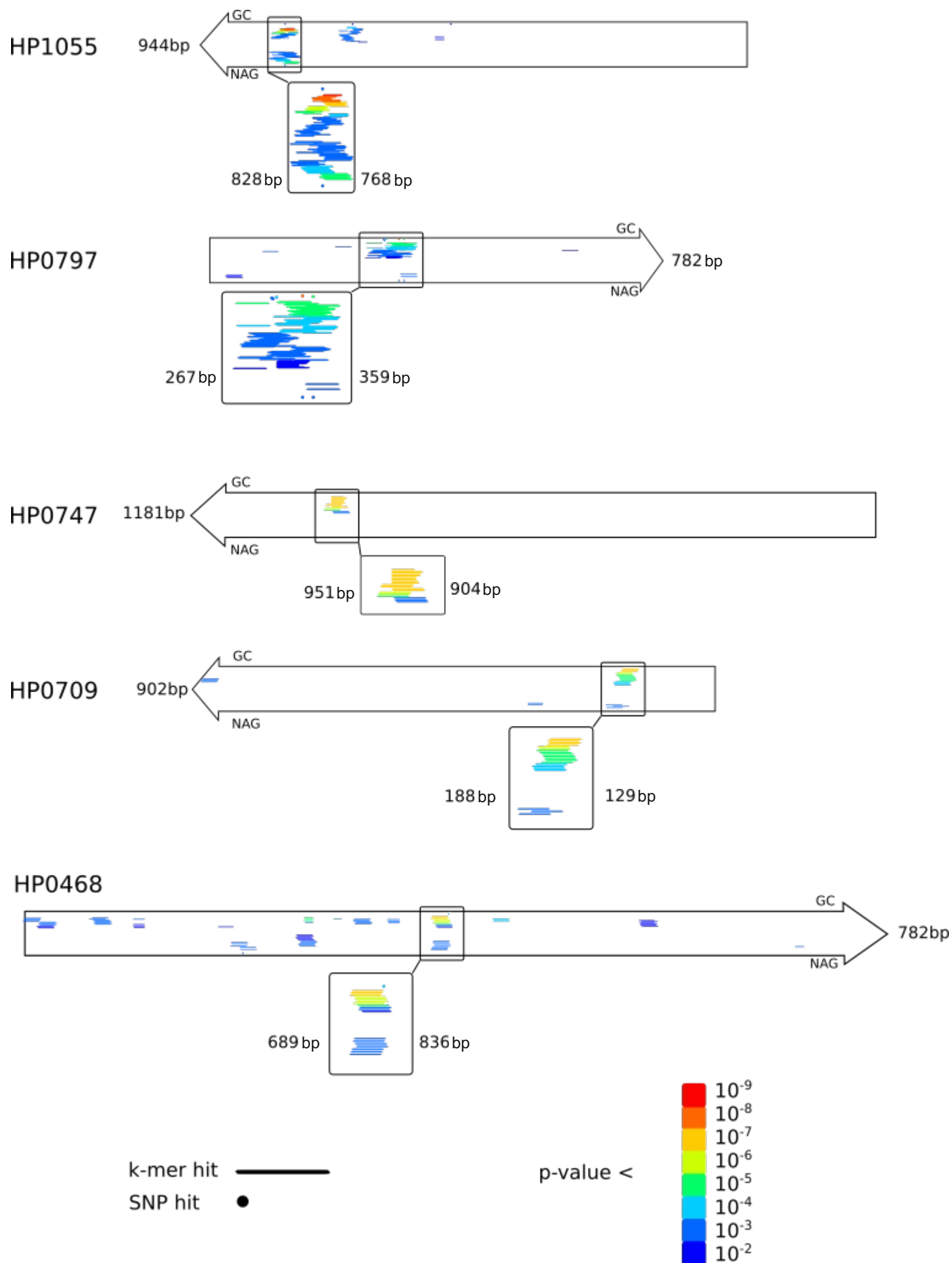

Supplement: Supplementary file 3 — Figure S3. Representation of the GWAS hits in the 5 non-accessory genes used in calculation of a risk score. All the hits with a p value < 0.05 are represented in the figure. The direction of the arrow representing the gene indicates on which strand the gene was found in ELS37 genome, and the length of each arrow is proportional to the length of the ELS37 version of the gene. Hits are positioned on the genes according to their position in ELS37 version of the genes. In each gene, the top half represents hits in the GC vs rest GWAS, and the bottom half represents hits in the NAG vs rest GWAS. K-mer hits are represented as lines, and SNP hits are represented as dots. Zoomed areas correspond to the areas where the genomic variations used in the risk score were found. (PDF 1523 kb) [file 12915_2018_550_MOESM3_ESM.pdf]

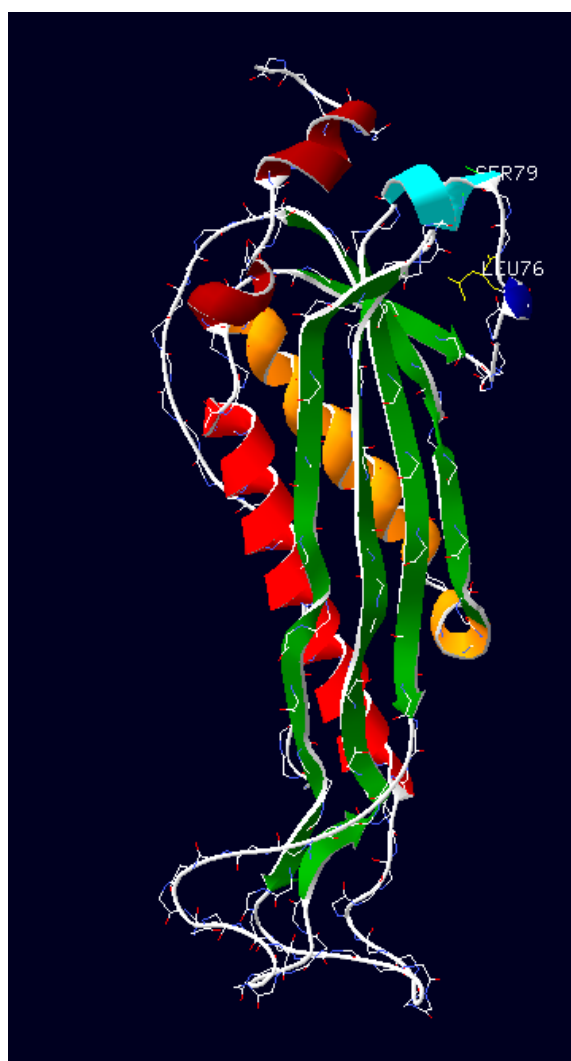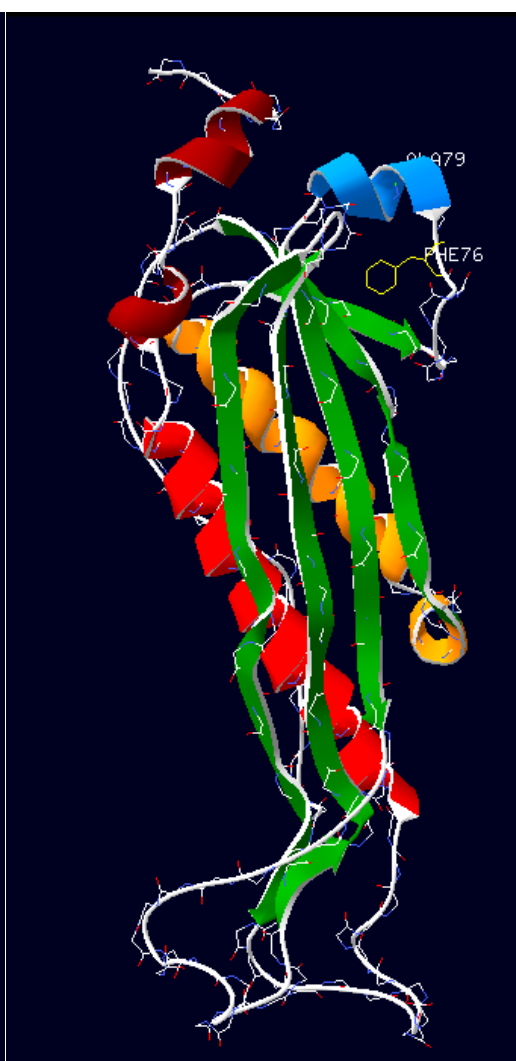

Supplement: Supplementary file 4 — Figure S4. 3D renderings of the safe and risk allele of HpaA. The 3D structure of 26,695 amino acid sequence of HpaA (HP0797) containing the safe allele (A) and the risk allele (B) respectively was modelled using 2I9I as template. Note that the helix formations in the area changes due to the mutations. Safe allele on the right with Leu 109 and Ser 112 (first 36 aa not included in model) and risk allele to the left with Phe 109 and Ala 112. (PDF 103 kb) [file 12915_2018_550_MOESM4_ESM.pdf]
