# Supplementary material for: A GWAS on Helicobacter pylori strains points to genetic variants associated with gastric cancer risk
Source: BMC Biol. 2018 Aug 2;16:84. doi: 10.1186/s12915-018-0550-3 (PMC6090961; doi:10.1186/s12915-018-0550-3)
Supplement: Supplementary file 5 — Table S1. Isolate details for the global 565 strains dataset. Summary of geographic provenance, fineSTRUCTURE population and source for the global dataset of 565 strains. Table S2. Isolate details for the hpEurope GWAS dataset. Summary of metadata for the 173 strains used in the GWAS study. Host pathology, GWAS group, isolation country, isolation city or region and H. pylori population are given when available. Table S3. List of the 32 genes highlighted in at least one of the GWAS experiments. The minimum p value and an annotation (obtained by Prokka) is mentioned for each gene. (DOCX 89 kb) [file 12915_2018_550_MOESM5_ESM.docx]

| **Table S1, Isolate details for the global 565 strains dataset. (page 1)** | | | | | | | | | | | | | | | | |  |  |
| --- | --- | --- | --- | --- | --- | --- | --- | --- | --- | --- | --- | --- | --- | --- | --- | --- | --- | --- |
| **Isolate** | | **Geographic provenance** | | | | **FineStructure Population** | | | | | | **Source** | | | | | |  |
| 26695_Tomb | | United Kingdom | | | | hspAfrica2 | | | | | | GCA_000008525.1 | | | | | | |
| Puno135 | | Puno, Peru | | | | hspAfrica2 | | | | | | GCA_000224555.1 | | | | | | |
| Gambia94/24 | | Gambia | | | | hspEAsia | | | | | | GCA_000185205.1 | | | | | | |
| B45 | | France | | | | hspEAsia | | | | | | GCA_000234645.4 | | | | | | |
| 52 | | Korea | | | | hspEuropeN | | | | | | GCA_000023805.1 | | | | | | |
| 2018 | | West Africa | | | | hspEAsia | | | | | | GCA_000192335.1 | | | | | | |
| 35A | | Japan | | | | hspEAsia | | | | | | GCA_000178935.2 | | | | | | |
| 51 | | South Korea | | | | hspEAsia | | | | | | GCA_000011725.1 | | | | | | |
| 83 | | Unknown | | | | hpAsia2 | | | | | | GCA_000213135.1 | | | | | | |
| Aklavik117 | | Aklavik, Canada | | | | hspEAsia | | | | | | GCA_000315955.1 | | | | | | |
| Aklavik86 | | Aklavik, Canada | | | | hpAsia2 | | | | | | GCA_000317875.1 | | | | | | |
| B38 | | France | | | | hspEAsia | | | | | | GCA_000091345.1 | | | | | | |
| Cuz20 | | Cuzco, Peru | | | | hspEAsia | | | | | | GCA_000148895.1 | | | | | | |
| ELS37 | | El Salvador | | | | hpAsia2 | | | | | | GCA_000255955.1 | | | | | | |
| F16 | | Japan | | | | hspAfrica1NAmerica | | | | | | GCA_000270005.1 | | | | | | |
| F30 | | Japan | | | | hspAfrica1NAmerica | | | | | | GCA_000270025.1 | | | | | | |
| F32 | | Japan | | | | hspEuropeColombia | | | | | | GCA_000270045.1 | | | | | | |
| F57 | | Japan | | | | hspEuropeColombia | | | | | | GCA_000270065.1 | | | | | | |
| G27 | | Italy | | | | hspEuropeColombia | | | | | | GCA_000021165.1 | | | | | | |
| HPAG1 | | Sweden | | | | hspEuropeColombia | | | | | | GCA_000013245.1 | | | | | | |
| HUP-B14 | | Spain | | | | hspAfrica1WAfrica | | | | | | GCA_000259235.1 | | | | | | |
| India7 | | India | | | | hpAsia2 | | | | | | GCA_000185185.1 | | | | | | |
| J99 | | USA | | | | hspAfrica1SAfrica | | | | | | GCA_000008785.1 | | | | | | |
| Lithuania75 | | Lithuania | | | | hspAfrica2 | | | | | | GCA_000185225.1 | | | | | | |
| OK113 | | Japan | | | | hspAfrica1SAfrica | | | | | | GCA_000348865.1 | | | | | | |
| OK310 | | Japan | | | | hspAfrica2 | | | | | | GCA_000348885.1 | | | | | | |
| P12 | | Germany | | | | hspAfrica1SAfrica | | | | | | GCA_000021465.1 | | | | | | |
| PeCan18 | | Lima, Peru | | | | hspAfrica1SAfrica | | | | | | GCA_000277425.1 | | | | | | |
| PeCan4 | | Peru | | | | hspAfrica1SAfrica | | | | | | GCA_000148875.1 | | | | | | |
| Puno120 | | Puno, Peru | | | | hspAfrica1SAfrica | | | | | | GCA_000224535.1 | | | | | | |
| SJM180 | | Lima, Peru | | | | hspEuropeN | | | | | | GCA_000148855.1 | | | | | | |
| **Table S1, Isolate details for the global 565 strains dataset. (page 2)** | | | | | | | | | | | | | | | | |  |  |
| **Isolate** | | | **Geographic provenance** | | | | | **FineStructure Population** | | | | | **Source** | | | | |  |
| SNT49 | | | Santal tribe in India | | | | | | hspAfrica2 | | | | GCA_000224575.1 | | | | | |
| Sat464 | | | Satipo region, Peru | | | | | | hspAfrica1SAfrica | | | | GCA_000148915.1 | | | | | |
| Shi112 | | | Shimaa village, Peru | | | | | | hspAfrica2 | | | | GCA_000277405.1 | | | | | |
| Shi169 | | | Shimaa village, Peru | | | | | | hspAfrica1SAfrica | | | | GCA_000277385.1 | | | | | |
| Shi417 | | | Shimaa village, Peru | | | | | | hspEuropeN | | | | GCA_000277365.1 | | | | | |
| Shi470 | | | Shimaa village, Peru | | | | | | hspEuropeN | | | | GCA_000020245.1 | | | | | |
| SouthAfrica7 | | | Bantu, Soweto, SA | | | | | | hspAfrica2 | | | | GCA_000185245.1 | | | | | |
| XZ274 | | | Tibet | | | | | | hspAfrica2 | | | | GCA_000262655.1 | | | | | |
| v225d | | | Piaroan Indian, Venezuela | | | | | | hspAfrica2 | | | | GCA_000093185.1 | | | | | |
| FD423 | | | Indian origin, Malaysia/India | | | | | | hspEuropeN | | | | GCA_000281675.2 | | | | | |
| FD430 | | | Indian origin, Malaysia/India | | | | | | hspAfrica1SAfrica | | | | GCA_000295975.2 | | | | | |
| FD506 | | | Chinese origin, Malaysia/Chinese | | | | | | hspAfrica1SAfrica | | | | GCA_000295995.2 | | | | | |
| FD535 | | | Indian origin, Malaysia/India | | | | | | hspEuropeN | | | | GCA_000296015.2 | | | | | |
| FD568 | | | Chinese origin, Malaysia/Chinese | | | | | | hpAsia2 | | | | GCA_000296035.2 | | | | | |
| FD577 | | | Chinese origin, Malaysia/Chinese | | | | | | hspAfrica1SAfrica | | | | GCA_000296055.2 | | | | | |
| FD703 | | | Malaysian | | | | | | hspAfrica1SAfrica | | | | GCA_000296075.2 | | | | | |
| FD662 | | | Malaysian | | | | | | hspAfrica2 | | | | GCA_000296095.2 | | | | | |
| FD719 | | | Malaysian | | | | | | hspAfrica2 | | | | GCA_000296115.2 | | | | | |
| GC26 | | | Chinese origin, Malaysia/Chinese | | | | | | hspAfrica2 | | | | GCA_000296135.2 | | | | | |
| 8A3 | | | Unknown | | | | | | hspAfrica1SAfrica | | | | GCA_000285055.1 | | | | | |
| 98-10 | | | Japan | | | | | | hspEuropeN | | | | GCA_000172935.1 | | | | | |
| A45 | | | Moscow, Russia | | | | | | hspAfrica2 | | | | GCA_000333835.1 | | | | | |
| B128 | | | USA (Info from BIGSdb only) | | | | | | hspAfrica1SAfrica | | | | GCA_000172955.1 | | | | | |
| CCHI33 | | | Canada | | | | | | hspAfrica2 | | | | GCA_000349485.1 | | | | | |
| CPY1124 | | | Japan | | | | | | hspAfrica1SAfrica | | | | GCA_000275225.2 | | | | | |
| CPY1313 | | | Japan | | | | | | hspAfrica2 | | | | GCA_000275205.1 | | | | | |
| CPY1662 | | | Japan | | | | | | hspEuropeN | | | | GCA_000349505.1 | | | | | |
| CPY1962 | | | Japan | | | | | | hspAfrica2 | | | | GCA_000275185.1 | | | | | |
| CPY3281 | | | Japan | | | | | | hspAfrica1SAfrica | | | | GCA_000274725.1 | | | | | |
| CPY6081 | | | Japan | | | | | | hspAfrica1SAfrica | | | | GCA_000274705.1 | | | | | |
| CPY6261 | | | Japan | | | | | | hspAfrica2 | | | | GCA_000274685.1 | | | | | |
| **Table S1, Isolate details for the global 565 strains dataset. (page 3)** | | | | | | | | | | | | | | | | |  |  |
| **Isolate** | | | | **Geographic provenance** | | | | | **FineStructure Population** | | | | | **Source** | | | |  |
| CPY6271 | | | | Japan | | | | | hspAfrica2 | | | | | GCA_000274665.1 | | | | |
| CPY6311 | | | | Japan | | | | | hspEuropeN | | | | | GCA_000274645.2 | | | | |
| GAM100Ai | | | | Gambia | | | | | hspAmerind | | | | | GCA_000310005.2 | | | | |
| GAM101Biv | | | | Gambia | | | | | hspAmerind | | | | | GCA_000344945.2 | | | | |
| GAM103Bi | | | | Gambia | | | | | hspEuropeS | | | | | GCA_000344965.2 | | | | |
| GAM105Ai | | | | Gambia | | | | | hspEuropeN | | | | | GCA_000344985.2 | | | | |
| GAM112Ai | | | | Gambia | | | | | hspEAsia | | | | | GCA_000345005.2 | | | | |
| GAM114Ai | | | | Gambia | | | | | hspEAsia | | | | | GCA_000345025.2 | | | | |
| GAM115Ai | | | | Gambia | | | | | hspEuropeS | | | | | GCA_000345045.2 | | | | |
| GAM118Bi | | | | Gambia | | | | | hspEuropeN | | | | | GCA_000345085.2 | | | | |
| GAM119Bi | | | | Gambia | | | | | hspEuropeS | | | | | GCA_000345105.2 | | | | |
| GAM120Ai | | | | Gambia | | | | | hspEuropeS | | | | | GCA_000345125.2 | | | | |
| GAM121Aii | | | | Gambia | | | | | hspEuropeN | | | | | GCA_000345145.2 | | | | |
| GAM201Ai | | | | Gambia | | | | | hspEuropeN | | | | | GCA_000345065.2 | | | | |
| GAM210Bi | | | | Gambia | | | | | hspEuropeS | | | | | GCA_000345165.2 | | | | |
| GAM231Ai | | | | Gambia | | | | | hspEuropeN | | | | | GCA_000345185.2 | | | | |
| GAM239Bi | | | | Gambia | | | | | hspEuropeS | | | | | GCA_000345205.2 | | | | |
| GAM244Ai | | | | Gambia | | | | | hspEuropeN | | | | | GCA_000345225.2 | | | | |
| GAM245Ai | | | | Gambia | | | | | hspAfrica1NAmerica | | | | | GCA_000345245.2 | | | | |
| GAM246Ai | | | | Gambia | | | | | hspEuropeS | | | | | GCA_000345265.2 | | | | |
| GAM249T | | | | Gambia | | | | | hspEuropeN | | | | | GCA_000345285.2 | | | | |
| GAM250AFi | | | | Gambia | | | | | hspEuropeN | | | | | GCA_000345305.2 | | | | |
| GAM254Ai | | | | Gambia | | | | | hspEuropeN | | | | | GCA_000345385.2 | | | | |
| GAM260ASi | | | | Gambia | | | | | hspEuropeN | | | | | GCA_000345405.2 | | | | |
| GAM260BSi | | | | Gambia | | | | | hspEuropeN | | | | | GCA_000345445.2 | | | | |
| GAM260Bi | | | | Gambia | | | | | hspEuropeN | | | | | GCA_000345425.2 | | | | |
| GAM263BFi | | | | Gambia | | | | | hspEuropeN | | | | | GCA_000345465.2 | | | | |
| GAM264Ai | | | | Gambia | | | | | hspEuropeN | | | | | GCA_000345485.2 | | | | |
| GAM265BSii | | | | Gambia | | | | | hspEuropeN | | | | | GCA_000345505.2 | | | | |
| GAM270ASi | | | | Gambia | | | | | hspEuropeN | | | | | GCA_000345545.2 | | | | |
| GAM71Ai | | | | Gambia | | | | | hspEuropeN | | | | | GCA_000345585.2 | | | | |
| **Table S1, Isolate details for the global 565 strains dataset. (page 4)** | | | | | | | | | | | | | | | | |  |  |
| **Isolate** | | | | | **Geographic provenance** | | | | **FineStructure Population** | | | | | **Source** | | | |  |
| GAM80Ai | | | | | Gambia | | | | hspEuropeS | | | | | | GCA_000345605.2 | | | |
| GAM83Bi | | | | | Gambia | | | | hspEuropeN | | | | | | GCA_000345625.2 | | | |
| GAM93Bi | | | | | Gambia | | | | hspEuropeN | | | | | | GCA_000345665.2 | | | |
| GAM96Ai | | | | | Gambia | | | | hspEuropeN | | | | | | GCA_000345685.2 | | | |
| GAMchjs106B | | | | | Gambia | | | | hspEuropeN | | | | | | GCA_000345705.2 | | | |
| GAMchjs114i | | | | | Gambia | | | | hspEuropeN | | | | | | GCA_000346815.2 | | | |
| GAMchjs117Ai | | | | | Gambia | | | | hspEuropeN | | | | | | GCA_000346835.2 | | | |
| GAMchjs124i | | | | | Gambia | | | | hspEuropeN | | | | | | GCA_000346855.2 | | | |
| GAMchjs136i | | | | | Gambia | | | | hspEuropeN | | | | | | GCA_000346875.2 | | | |
| HLJHP193 | | | | | China | | | | hspEuropeN | | | | | | GCA_000287835.1 | | | |
| HLJHP253 | | | | | China | | | | hspEuropeN | | | | | | GCA_000287775.1 | | | |
| HLJHP256 | | | | | China | | | | hspEuropeN | | | | | | GCA_000287735.1 | | | |
| HLJHP271 | | | | | China | | | | hspEuropeN | | | | | | GCA_000287755.1 | | | |
| HP116Bi | | | | | Gambia | | | | hspEuropeN | | | | | | GCA_000345725.2 | | | |
| A-11 | | | | | USA | | | | hspEuropeN | | | | | | GCA_000359645.1 | | | |
| A-14 | | | | | USA | | | | hspEuropeN | | | | | | GCA_000274005.2 | | | |
| A-16 | | | | | USA | | | | hspEuropeN | | | | | | GCA_000275165.1 | | | |
| A-17 | | | | | USA | | | | hspEuropeN | | | | | | GCA_000275265.2 | | | |
| A-20 | | | | | USA | | | | hspEuropeN | | | | | | GCA_000275285.1 | | | |
| A-26 | | | | | USA | | | | hspEuropeS | | | | | | GCA_000274025.1 | | | |
| A-27 | | | | | USA | | | | hspEuropeN | | | | | | GCA_000274045.2 | | | |
| Hp-_A-4 | | | | | USA | | | | hspEuropeS | | | | | | GCA_000275305.2 | | | |
| Hp-A-5 | | | | | USA | | | | hspEuropeN | | | | | | GCA_000275045.1 | | | |
| Hp-A-6 | | | | | USA | | | | hspEuropeN | | | | | | GCA_000273985.1 | | | |
| A-8 | | | | | USA | | | | hspEuropeN | | | | | | GCA_000273825.1 | | | |
| Hp-_A-9 | | | | | Cleveland, OH, USA | | | | hspEuropeS | | | | | | GCA_000275065.2 | | | |
| H-1 | | | | | USA | | | | hspEuropeN | | | | | | GCA_000349405.1 | | | |
| H-10 | | | | | Cleveland, OH, USA | | | | hspEuropeN | | | | | | GCA_000275325.1 | | | |
| H-11 | | | | | USA | | | | hspEuropeS | | | | | | GCA_000274485.2 | | | |
| H-16 | | | | | USA | | | | hspAfrica1SAfrica | | | | | | GCA_000275245.2 | | | |
| H-18 | | | | | Cleveland, OH, USA | | | | hspAfrica1SAfrica | | | | | | GCA_000274505.1 | | | |
| **Table S1, Isolate details for the global 565 strains dataset. (page 5)** | | | | | | | | | | | | | | | | |  |  |
| **Isolate** | | | | | **Geographic provenance** | | | | **FineStructure Population** | | | | | | | **Source** | |  |
| H-19 | | | | | USA | | | | hspEuropeS | | | | | | | GCA_000274125.1 | | |
| H-21 | | | | | USA | | | | hspAfrica1WAfrica | | | | | | | GCA_000274185.2 | | |
| H-23 | | | | | USA | | | | hspAfrica1NAmerica | | | | | | | GCA_000274165.1 | | |
| H-24 | | | | | USA | | | | hspEAsia | | | | | | | GCA_000275085.1 | | |
| H-27 | | | | | USA | | | | hspEuropeS | | | | | | | GCA_000275105.1 | | |
| H-28 | | | | | Cleveland, OH, USA | | | | hpAsia2 | | | | | | | GCA_000275125.1 | | |
| H-29 | | | | | Cleveland, OH, USA | | | | hspEuropeN | | | | | | | GCA_000273885.1 | | |
| H-3 | | | | | USA | | | | hspEuropeS | | | | | | | GCA_000274065.1 | | |
| H-30 | | | | | USA | | | | hspEuropeS | | | | | | | GCA_000273865.2 | | |
| H-34 | | | | | USA | | | | hspEuropeS | | | | | | | GCA_000274265.1 | | |
| H-36 | | | | | Cleveland, OH, USA | | | | hspEuropeN | | | | | | | GCA_000273845.1 | | |
| H-4 | | | | | USA | | | | hspEuropeN | | | | | | | GCA_000274145.1 | | |
| H-41 | | | | | USA | | | | hspEuropeS | | | | | | | GCA_000273805.1 | | |
| H-42 | | | | | Cleveland, OH, USA | | | | hspEuropeS | | | | | | | GCA_000273905.1 | | |
| H-43 | | | | | USA | | | | hspEuropeS | | | | | | | GCA_000273965.1 | | |
| H-44 | | | | | Cleveland, OH, USA | | | | hspEuropeS | | | | | | | GCA_000273925.1 | | |
| H-45 | | | | | USA | | | | hspEuropeS | | | | | | | GCA_000273945.1 | | |
| H-5b | | | | | Cleveland, OH, USA | | | | hspEAsia | | | | | | | GCA_000274365.1 | | |
| H-6 | | | | | USA | | | | hpAsia2 | | | | | | | GCA_000274105.1 | | |
| H-9 | | | | | USA | | | | hspEAsia | | | | | | | GCA_000274085.2 | | |
| P-1 | | | | | USA | | | | hpAsia2 | | | | | | | GCA_000274785.1 | | |
| P-11 | | | | | USA | | | | hpAsia2 | | | | | | | GCA_000274205.2 | | |
| P-13 | | | | | USA | | | | hpAsia2 | | | | | | | GCA_000274225.1 | | |
| P-15 | | | | | USA | | | | hpAsia2 | | | | | | | GCA_000274285.1 | | |
| P-16 | | | | | USA | | | | hspEAsia | | | | | | | GCA_000274305.1 | | |
| P-2 | | | | | USA | | | | hspEAsia | | | | | | | GCA_000274805.1 | | |
| P-23 | | | | | USA | | | | hpAsia2 | | | | | | | GCA_000274325.1 | | |
| P-25 | | | | | USA | | | | hpAsia2 | | | | | | | GCA_000274865.1 | | |
| P-26 | | | | | USA | | | | hpAsia2 | | | | | | | GCA_000275385.1 | | |
| P-28b | | | | | USA | | | | hpAsia2 | | | | | | | GCA_000275345.2 | | |
| P-3 | | | | | USA | | | | hspEuropeN | | | | | | | GCA_000274825.2 | | |
| **Table S1, Isolate details for the global 565 strains dataset. (page 6)** | | | | | | | | | | | | | | | | |  |  |
| **Isolate** | | | | | **Geographic provenance** | | | | | **FineStructure Population** | | | | | | **Source** | |  |
| P-30 | | | | | USA | | | | | hpAsia2 | | | | | | GCA_000275405.2 | | |
| P-4 | | | | | USA | | | | | hpAsia2 | | | | | | GCA_000274845.2 | | |
| P-41 | | | | | USA | | | | | hpAsia2 | | | | | | GCA_000275425.2 | | |
| P-62 | | | | | USA | | | | | hspEuropeN | | | | | | GCA_000275445.2 | | |
| P-74 | | | | | USA | | | | | hpAsia2 | | | | | | GCA_000274345.1 | | |
| P-8 | | | | | USA | | | | | hpAsia2 | | | | | | GCA_000274245.1 | | |
| N6 | | | | | France | | | | | hpAsia2 | | | | | | GCA_000285895.1 | | |
| NAB47 | | | | | Bangalore, India | | | | | hpAsia2 | | | | | | GCA_000256075.2 | | |
| NAD1 | | | | | Delhi, India | | | | | hpAsia2 | | | | | | GCA_000256035.2 | | |
| NCTC_11637 | | | | | Australia | | | | | hpAsia2 | | | | | | GCA_000258845.1 | | |
| NQ1671 | | | | | Narino, Colombia | | | | | hpAsia2 | | | | | | GCA_000285235.1 | | |
| NQ1701 | | | | | Narino, Colombia | | | | | hpAsia2 | | | | | | GCA_000285135.1 | | |
| NQ4044 | | | | | Colombia | | | | | hpAsia2 | | | | | | GCA_000274625.1 | | |
| NQ4053 | | | | | Colombia | | | | | hpAsia2 | | | | | | GCA_000274605.1 | | |
| NQ4076 | | | | | Colombia | | | | | hpAsia2 | | | | | | GCA_000274585.1 | | |
| NQ4099 | | | | | Colombia | | | | | hpAsia2 | | | | | | GCA_000274565.1 | | |
| NQ4110 | | | | | Colombia | | | | | hpAsia2 | | | | | | GCA_000274545.2 | | |
| NQ4161 | | | | | Colombia | | | | | hspEuropeN | | | | | | GCA_000274525.2 | | |
| NQ4200 | | | | | Colombia | | | | | hspEAsia | | | | | | GCA_000274905.1 | | |
| NQ4216 | | | | | Columbia | | | | | hpAsia2 | | | | | | GCA_000274945.2 | | |
| NQ4228 | | | | | Colombia | | | | | hspEuropeN | | | | | | GCA_000274925.1 | | |
| R018c | | | | | Canada | | | | | hpAsia2 | | | | | | GCA_000299855.1 | | |
| R030b | | | | | Canada | | | | | hpAsia2 | | | | | | GCA_000299695.1 | | |
| R036d | | | | | Canada | | | | | hspEAsia | | | | | | GCA_000299715.1 | | |
| R037c | | | | | Canada | | | | | hspEAsia | | | | | | GCA_000299735.1 | | |
| R038b | | | | | Canada | | | | | hspEAsia | | | | | | GCA_000299755.1 | | |
| R046Wa | | | | | Canada | | | | | hspEAsia | | | | | | GCA_000299775.1 | | |
| R055a | | | | | Canada | | | | | hpAsia2 | | | | | | GCA_000299795.1 | | |
| R32b | | | | | Canada | | | | | hspEuropeN | | | | | | GCA_000299835.1 | | |
| UMB_G1 | | | | | Canada | | | | | hspEuropeN | | | | | | GCA_000349465.1 | | |
| UM007 | | | | | Malaysia | | | | | hspEAsia | | | | | | GCA_000401355.1 | | |
| **Table S1, Isolate details for the global 565 strains dataset. (page 7)** | | | | | | | | | | | | | | | | |  |  |
| **Isolate** | | | | | **Geographic provenance** | | | | | **FineStructure Population** | | | | | | **Source** | |  |
| UM018 | | | | | Malaysia | | | | | hspEuropeN | | | | | | GCA_000401315.1 | | |
| UM034 | | | | | Malaysia | | | | | hspEuropeS | | | | | | GCA_000401375.1 | | |
| UM045 | | | | | Malaysia | | | | | hspAfrica1Americas | | | | | | GCA_001051495.1 | | |
| UM054 | | | | | Malaysia | | | | | hspAfrica1Americas | | | | | | GCA_000401335.1 | | |
| SouthAfrica20 | | | | | Bantu, Soweto, SA | | | | | hspAfrica1Americas | | | | | | GCA_000590775.1 | | |
| SouthAfrica50 | | | | | Bantu, Soweto, SA | | | | | hspAfrica1Americas | | | | | | GCA_000448525.1 | | |
| UM023 | | | | | Chinese origin, Malaysia/Chinese | | | | | hspAfrica1Americas | | | | | | GCA_000444325.1 | | |
| UM032 | | | | | Kuala Lumpur, Malaysia | | | | | hspAfrica1Americas | | | | | | GCA_000392455.3 | | |
| UM037 | | | | | Indian origin, Malaysia/India | | | | | hspEuropeN | | | | | | GCA_000451005.1 | | |
| UM038 | | | | | Chinese origin, Malaysia/Chinese | | | | | hspEuropeS | | | | | | GCA_000444345.1 | | |
| UM065 | | | | | Chinese origin, Malaysia/Chinese | | | | | hspAfrica1Americas | | | | | | GCA_000444365.1 | | |
| UM066 | | | | | Chinese origin, Malaysia/Chinese | | | | | hspAfrica1Americas | | | | | | GCA_000451025.1 | | |
| UM067 | | | | | Indian origin, Malaysia/India | | | | | hspAfrica1Americas | | | | | | GCA_000444785.1 | | |
| UM077 | | | | | Chinese origin, Malaysia/Chinese | | | | | hspAfrica1Americas | | | | | | GCA_000444725.1 | | |
| UM084 | | | | | Malaysia | | | | | hspAfrica1Americas | | | | | | GCA_000444385.1 | | |
| UM085 | | | | | Chinese origin, Malaysia/Chinese | | | | | hspAfrica1Americas | | | | | | GCA_000444745.1 | | |
| UM111 | | | | | Chinese origin, Malaysia/Chinese | | | | | hspEuropeS | | | | | | GCA_000444305.1 | | |
| UM114 | | | | | Indian origin, Malaysia/India | | | | | hspEuropeS | | | | | | GCA_000444495.1 | | |
| PZ5004 | | | | | Tumaco, Colombia (Low GC Risk) | | | | | hspAfrica1Americas | | | | | | GCA_000448405.1 | | |
| PZ5024 | | | | | Tumaco, Colombia (Low GC Risk) | | | | | hspEuropeS | | | | | | GCA_000448425.1 | | |
| PZ5026 | | | | | Tumaco, Colombia (Low GC Risk) | | | | | hspAfrica1Americas | | | | | | GCA_000448445.1 | | |
| PZ5056 | | | | | Tuquerres, Colombia (High GC risk) | | | | | hspAfrica1Americas | | | | | | GCA_000448465.1 | | |
| PZ5080 | | | | | Tuquerres, Colombia (High GC risk) | | | | | hspAfrica1Americas | | | | | | GCA_000448485.1 | | |
| PZ5086 | | | | | Tuquerres, Colombia (High GC risk) | | | | | hspEuropeS | | | | | | GCA_000448505.1 | | |
| GAM117Ai | | | | | Gambia | | | | | hspAfrica1NAmerica | | | | | | GCA_000455765.1 | | |
| NAK7 | | | | | India | | | | | hspAfrica1Americas | | | | | | GCA_000401295.1 | | |
| SA157A | | | | | Ogies, Mpumalanga, South Africa | | | | | hspEuropeColombia | | | | | | GCA_000475615.1 | | |
| SA144A | | | | | Ogies, Mpumalanga, South Africa | | | | | hspEuropeColombia | | | | | | GCA_000475715.1 | | |
| SA146A | | | | | Ogies, Mpumalanga, South Africa | | | | | hspEAsia | | | | | | GCA_000477175.1 | | |
| SA155A | | | | | Ogies, Mpumalanga, South Africa | | | | | hspEAsia | | | | | | GCA_000475795.1 | | |
| SA156A | | | | | Ogies, Mpumalanga, South Africa | | | | | hspEAsia | | | | | | GCA_000475775.1 | | |
| **Table S1, Isolate details for the global 565 strains dataset. (page 8)** | | | | | | | | | | | | | | | | |  |  |
| **Isolate** | | | | | **Geographic provenance** | | | | | **FineStructure Population** | | | | | | **Source** | |  |
| SA158A | | | | | Ogies, Mpumalanga, South Africa | | | | | hspEAsia | | | | | | GCA_000476075.1 | | |
| SA161A | | | | | Ogies, Mpumalanga, South Africa | | | | | hspAfrica1Americas | | | | | | GCA_000476395.1 | | |
| SA162A | | | | | Ogies, Mpumalanga, South Africa | | | | | hspAfrica1Americas | | | | | | GCA_000476115.1 | | |
| SA164A | | | | | Ogies, Mpumalanga, South Africa | | | | | hspAfrica1Americas | | | | | | GCA_000477115.1 | | |
| SA166A | | | | | Ogies, Mpumalanga, South Africa | | | | | hspEuropeS | | | | | | GCA_000476255.1 | | |
| SA168A | | | | | Ogies, Mpumalanga, South Africa | | | | | hspAfrica1Americas | | | | | | GCA_000476675.1 | | |
| SA169C | | | | | Ogies, Mpumalanga, South Africa | | | | | hspAfrica1Americas | | | | | | GCA_000475955.1 | | |
| SA170C | | | | | Ogies, Mpumalanga, South Africa | | | | | hspAfrica1Americas | | | | | | GCA_000477155.1 | | |
| SA171A | | | | | Ogies, Mpumalanga, South Africa | | | | | hspAfrica1Americas | | | | | | GCA_000476055.1 | | |
| SA173A | | | | | Ogies, Mpumalanga, South Africa | | | | | hspAfrica1Americas | | | | | | GCA_000476575.1 | | |
| Hp-SA174A | | | | | Ogies, Mpumalanga, South Africa | | | | | hspAfrica1Americas | | | | | | GCA_000477015.1 | | |
| SA175A | | | | | Ogies, Mpumalanga, South Africa | | | | | hspEuropeN | | | | | | GCA_000476895.1 | | |
| SA194A | | | | | Ogies, Mpumalanga, South Africa | | | | | hspAmerind | | | | | | GCA_000477055.1 | | |
| SA213A | | | | | Ogies, Mpumalanga, South Africa | | | | | hspAfrica1WAfrica | | | | | | GCA_000476275.1 | | |
| SA214A | | | | | Ogies, Mpumalanga, South Africa | | | | | hspEuropeS | | | | | | GCA_000476195.1 | | |
| SA220A | | | | | Ogies, Mpumalanga, South Africa | | | | | hspEAsia | | | | | | GCA_000475535.1 | | |
| SA221A | | | | | Ogies, Mpumalanga, South Africa | | | | | hspAfrica1NAmerica | | | | | | GCA_000477095.1 | | |
| SA222A | | | | | Ogies, Mpumalanga, South Africa | | | | | hspEAsia | | | | | | GCA_000477195.1 | | |
| SA226A | | | | | Ogies, Mpumalanga, South Africa | | | | | hspEAsia | | | | | | GCA_000475655.1 | | |
| SA227A | | | | | Ogies, Mpumalanga, South Africa | | | | | hspEAsia | | | | | | GCA_000476035.1 | | |
| SA233A | | | | | Ogies, Mpumalanga, South Africa | | | | | hspAmerind | | | | | | GCA_000475475.1 | | |
| SA253A | | | | | Ogies, Mpumalanga, South Africa | | | | | hspAmerind | | | | | | GCA_000476235.1 | | |
| SA29A | | | | | Ogies, Mpumalanga, South Africa | | | | | hspEuropeN | | | | | | GCA_000476535.1 | | |
| SA301A | | | | | Ogies, Mpumalanga, South Africa | | | | | hspAmerind | | | | | | GCA_000476715.1 | | |
| SA302A | | | | | Ogies, Mpumalanga, South Africa | | | | | hspEuropeS | | | | | | GCA_000476175.1 | | |
| SA303C | | | | | Ogies, Mpumalanga, South Africa | | | | | hspEAsia | | | | | | GCA_000476655.1 | | |
| SA30A | | | | | Ogies, Mpumalanga, South Africa | | | | | hspEAsia | | | | | | GCA_000476635.1 | | |
| SA34A | | | | | Ogies, Mpumalanga, South Africa | | | | | hspEAsia | | | | | | GCA_000476415.1 | | |
| SA35A | | | | | Ogies, Mpumalanga, South Africa | | | | | hspEAsia | | | | | | GCA_000475495.1 | | |
| SA36C | | | | | Ogies, Mpumalanga, South Africa | | | | | hspEuropeN | | | | | | GCA_000476995.1 | | |
| SA37A | | | | | Ogies, Mpumalanga, South Africa | | | | | hspEuropeN | | | | | | GCA_000476015.1 | | |
| **Table S1, Isolate details for the global 565 strains dataset. (page 9)** | | | | | | | | | | | | | | | | |  |  |
| **Isolate** | | | | | **Geographic provenance** | | | | | **FineStructure Population** | | | | | | **Source** | |  |
| SA40A | | | | | Ogies, Mpumalanga, South Africa | | | | | hspEuropeS | | | | | GCA_000475735.1 | | | |
| SA45A | | | | | Ogies, Mpumalanga, South Africa | | | | | hpAsia2 | | | | | GCA_000475455.1 | | | |
| SA46C | | | | | Ogies, Mpumalanga, South Africa | | | | | hspAfrica1NAmerica | | | | | GCA_000475875.1 | | | |
| SA47A | | | | | Ogies, Mpumalanga, South Africa | | | | | hspEuropeN | | | | | GCA_000476295.1 | | | |
| SA160A | | | | | Ogies, Mpumalanga, South Africa | | | | | hspEAsia | | | | | GCA_000477235.1 | | | |
| Sahul64 | | | | | Western Australia (indigene) | | | | | hspEAsia | | | | | GCA_000513515.1 | | | |
| E48 | | | | | Russia: Evenk automous region | | | | | hspEuropeN | | | | | GCA_000499345.1 | | | |
| H13-1 | | | | | Habarovsk (Russia) | | | | | hspAfrica1Americas | | | | | GCA_000499325.1 | | | |
| HPARG63 | | | | | Unknown | | | | | hspAmerind | | | | | GCA_000438495.1 | | | |
| HPARG8G | | | | | Unknown | | | | | hspAmerind | | | | | GCA_000438475.1 | | | |
| wls-5-12 | | | | | China: Zhejiang | | | | | hspEuropeS | | | | | GCA_000444195.2 | | | |
| wls-5-3 | | | | | China: Zhejiang | | | | | hpAsia2 | | | | | GCA_000444175.1 | | | |
| B23 | | | | | Bordeaux, France | | | | | hspAmerind | | | | | SAMN08381202 | | | |
| B24 | | | | | Bordeaux, France | | | | | hspAmerind | | | | | SAMN08381203 | | | |
| B25 | | | | | Bordeaux, France | | | | | hspAmerind | | | | | SAMN08381204 | | | |
| B26 | | | | | Bordeaux, France | | | | | hspAmerind | | | | | SAMN08381205 | | | |
| B29 | | | | | Bordeaux, France | | | | | hspAmerind | | | | | SAMN08381206 | | | |
| B30 | | | | | Bordeaux, France | | | | | hspAfrica2 | | | | | SAMN08381207 | | | |
| B31 | | | | | Bordeaux, France | | | | | hspEAsia | | | | | SAMN08381208 | | | |
| B35 | | | | | Bordeaux, France | | | | | hspAmerind | | | | | SAMN08381209 | | | |
| B37 | | | | | Bordeaux, France | | | | | hpAsia2 | | | | | SAMN08381210 | | | |
| B40 | | | | | Bordeaux, France | | | | | hpAsia2 | | | | | SAMN08381211 | | | |
| B41 | | | | | Bordeaux, France | | | | | hspEAsia | | | | | SAMN08381212 | | | |
| B43 | | | | | Bordeaux, France | | | | | hpAsia2 | | | | | SAMN08381213 | | | |
| B44 | | | | | Bordeaux, France | | | | | hspEAsia | | | | | SAMN08381214 | | | |
| B47 | | | | | Bordeaux, France | | | | | hspEAsia | | | | | SAMN08381215 | | | |
| GC11-HL | | | | | Bordeaux, France | | | | | hpAsia2 | | | | | SAMN08381216 | | | |
| GC23-HL | | | | | Bordeaux, France | | | | | hpAsia2 | | | | | SAMN08381217 | | | |
| GC26-HL | | | | | Bordeaux, France | | | | | hpAsia2 | | | | | SAMN08381218 | | | |
| GC27-HL | | | | | Bordeaux, France | | | | | hspEAsia | | | | | SAMN08381219 | | | |
| GC31-B | | | | | Bordeaux, France | | | | | hspEAsia | | | | | SAMN08381220 | | | |
| **Table S1, Isolate details for the global 565 strains dataset. (page 10)** | | | | | | | | | | | | | | | | |  |  |
| **Isolate** | | | | | **Geographic provenance** | | | | | **FineStructure Population** | | | | | | **Source** | |  |
| GC34-HL | | | | | Bordeaux, France | | | | | hspEAsia | | | | | SAMN08381221 | | | |
| GC43-HL | | | | | Bordeaux, France | | | | | hpAsia2 | | | | | SAMN08381222 | | | |
| GC54-HL | | | | | Bordeaux, France | | | | | hspEuropeN | | | | | SAMN08381223 | | | |
| GC65-HL | | | | | Bordeaux, France | | | | | hspAfrica1NAmerica | | | | | SAMN08381224 | | | |
| GC67-HL | | | | | Bordeaux, France | | | | | hspEAsia | | | | | SAMN08381225 | | | |
| SSR1 | | | | | Dublin, Ireland | | | | | hspEAsia | | | | | SAMN08381226 | | | |
| SSR2 | | | | | Dublin, Ireland | | | | | hspEAsia | | | | | https://datadryad.org/resource/doi:10.5061/dryad.8qp4n/2 | | | |
| SSR3 | | | | | Dublin, Ireland | | | | | hspEAsia | | | | | https://datadryad.org/resource/doi:10.5061/dryad.8qp4n/2 | | | |
| SSR4 | | | | | Dublin, Ireland | | | | | hspEAsia | | | | | https://datadryad.org/resource/doi:10.5061/dryad.8qp4n/2 | | | |
| SSR5 | | | | | Dublin, Ireland | | | | | hspEAsia | | | | | https://datadryad.org/resource/doi:10.5061/dryad.8qp4n/2 | | | |
| SSR7 | | | | | Dublin, Ireland | | | | | hspEAsia | | | | | https://datadryad.org/resource/doi:10.5061/dryad.8qp4n/2 | | | |
| SSR8 | | | | | Dublin, Ireland | | | | | hspEAsia | | | | | https://datadryad.org/resource/doi:10.5061/dryad.8qp4n/2 | | | |
| SSR9 | | | | | Dublin, Ireland | | | | | hspEAsia | | | | | https://datadryad.org/resource/doi:10.5061/dryad.8qp4n/2 | | | |
| SSR12 | | | | | Dublin, Ireland | | | | | hspAfrica1WAfrica | | | | | https://datadryad.org/resource/doi:10.5061/dryad.8qp4n/2 | | | |
| SSR13 | | | | | Dublin, Ireland | | | | | hspAfrica1WAfrica | | | | | https://datadryad.org/resource/doi:10.5061/dryad.8qp4n/2 | | | |
| SSR14 | | | | | Dublin, Ireland | | | | | hspAfrica1WAfrica | | | | | https://datadryad.org/resource/doi:10.5061/dryad.8qp4n/2 | | | |
| SSR17 | | | | | Dublin, Ireland | | | | | hspAfrica1WAfrica | | | | | https://datadryad.org/resource/doi:10.5061/dryad.8qp4n/2 | | | |
| SSR20 | | | | | Dublin, Ireland | | | | | hspAfrica1WAfrica | | | | | https://datadryad.org/resource/doi:10.5061/dryad.8qp4n/2 | | | |
| SSR22 | | | | | Dublin, Ireland | | | | | hspAfrica1WAfrica | | | | | https://datadryad.org/resource/doi:10.5061/dryad.8qp4n/2 | | | |
| SSR23 | | | | | Dublin, Ireland | | | | | hspAfrica1WAfrica | | | | | https://datadryad.org/resource/doi:10.5061/dryad.8qp4n/2 | | | |
| SSR33 | | | | | Dublin, Ireland | | | | | hspAfrica1WAfrica | | | | | https://datadryad.org/resource/doi:10.5061/dryad.8qp4n/2 | | | |
| SSR40 | | | | | Dublin, Ireland | | | | | hspAfrica1WAfrica | | | | | https://datadryad.org/resource/doi:10.5061/dryad.8qp4n/2 | | | |
| SSR43 | | | | | Dublin, Ireland | | | | | hspAfrica1WAfrica | | | | | https://datadryad.org/resource/doi:10.5061/dryad.8qp4n/2 | | | |
| 3774 | | | | | Bordeaux, France | | | | | hspAfrica1WAfrica | | | | | SAMN08381227 | | | |
| 3843 | | | | | Bordeaux, France | | | | | hspAfrica1WAfrica | | | | | SAMN08381228 | | | |
| ANT_170 | | | | | Bordeaux, France | | | | | hspAfrica1NAmerica | | | | | SAMN08381229 | | | |
| GIL_237 | | | | | Bordeaux, France | | | | | hspAfrica1WAfrica | | | | | SAMN08381230 | | | |
| BON_254 | | | | | Bordeaux, France | | | | | hspAfrica1WAfrica | | | | | https://datadryad.org/resource/doi:10.5061/dryad.8qp4n/2 | | | |
| CHA_185 | | | | | Bordeaux, France | | | | | hspAfrica1WAfrica | | | | | https://datadryad.org/resource/doi:10.5061/dryad.8qp4n/2 | | | |
| GRA_247 | | | | | Bordeaux, France | | | | | hspAfrica1WAfrica | | | | | https://datadryad.org/resource/doi:10.5061/dryad.8qp4n/2 | | | |
| PHI_092 | | | | | Bordeaux, France | | | | | hspAfrica1WAfrica | | | | | https://datadryad.org/resource/doi:10.5061/dryad.8qp4n/2 | | | |
| **Table S1, Isolate details for the global 565 strains dataset. (page 11)** | | | | | | | | | | | | | | | | |  |  |
| **Isolate** | | **Geographic provenance** | | | | | **FineStructure Population** | | | | **Source** | | | | | | |  |
| GC30-HL | | Bordeaux, France | | | | | hspAfrica1WAfrica | | | | SAMN08381231 | | | | | | | |
| GC52-HL | | Bordeaux, France | | | | | hspAfrica1NAmerica | | | | SAMN08381232 | | | | | | | |
| 3755 | | Bordeaux, France | | | | | hspAfrica1WAfrica | | | | SAMN08381233 | | | | | | | |
| 3770 | | Bordeaux, France | | | | | hspAfrica1WAfrica | | | | SAMN08381234 | | | | | | | |
| 3800 | | Bordeaux, France | | | | | hspAfrica1WAfrica | | | | https://datadryad.org/resource/doi:10.5061/dryad.8qp4n/2 | | | | | | | |
| 3802 | | Bordeaux, France | | | | | hspAfrica1WAfrica | | | | SAMN08381235 | | | | | | | |
| 3824 | | Bordeaux, France | | | | | hspAfrica1WAfrica | | | | SAMN08381236 | | | | | | | |
| TN2GF4 | | Japan | | | | | hspAfrica1WAfrica | | | | SAMN08381237 | | | | | | | |
| 565/99 | | Amerindian strain | | | | | hspAfrica1WAfrica | | | | SAMN08381238 | | | | | | | |
| 3754 | | Bordeaux, France | | | | | hspAfrica1WAfrica | | | | https://datadryad.org/resource/doi:10.5061/dryad.8qp4n/2 | | | | | | | |
| 3745 | | Bordeaux, France | | | | | hspAfrica1WAfrica | | | | https://datadryad.org/resource/doi:10.5061/dryad.8qp4n/2 | | | | | | | |
| 3697 | | Bordeaux, France | | | | | hspAfrica1WAfrica | | | | https://datadryad.org/resource/doi:10.5061/dryad.8qp4n/2 | | | | | | | |
| 3738 | | Bordeaux, France | | | | | hspAfrica1WAfrica | | | | https://datadryad.org/resource/doi:10.5061/dryad.8qp4n/2 | | | | | | | |
| 3699 | | Bordeaux, France | | | | | hspAfrica1NAmerica | | | | SAMN08381239 | | | | | | | |
| 3746 | | Bordeaux, France | | | | | hspAfrica1WAfrica | | | | https://datadryad.org/resource/doi:10.5061/dryad.8qp4n/2 | | | | | | | |
| GC69-HL | | Bordeaux, France | | | | | hspAfrica1WAfrica | | | | SAMN08381240 | | | | | | | |
| 1152/04 | | Portugal (pediatric strain) | | | | | hspAfrica1WAfrica | | | | GCA_000802445.1 | | | | | | | |
| 1198/04 | | Portugal (pediatric strain) | | | | | hspAfrica1WAfrica | | | | GCA_000802465.1 | | | | | | | |
| 207/99 | | Portugal (pediatric strain) | | | | | hspAfrica1WAfrica | | | | GCA_000802505.1 | | | | | | | |
| 499/02 | | Portugal (pediatric strain) | | | | | hspAfrica1WAfrica | | | | GCA_000802525.1 | | | | | | | |
| 655/99 | | Portugal (pediatric strain) | | | | | hspEAsia | | | | GCA_000802575.1 | | | | | | | |
| Yangon244 | | Yangon, Myanmar | | | | | hspEAsia | | | | https://datadryad.org/resource/doi:10.5061/dryad.8qp4n/2 | | | | | | | |
| Yangon233 | | Yangon, Myanmar | | | | | hspEAsia | | | | https://datadryad.org/resource/doi:10.5061/dryad.8qp4n/2 | | | | | | | |
| Yangon222 | | Yangon, Myanmar | | | | | hspEAsia | | | | https://datadryad.org/resource/doi:10.5061/dryad.8qp4n/2 | | | | | | | |
| Yangon202 | | Yangon, Myanmar | | | | | hspAfrica1WAfrica | | | | https://datadryad.org/resource/doi:10.5061/dryad.8qp4n/2 | | | | | | | |
| Yangon190 | | Yangon, Myanmar | | | | | hspEuropeN | | | | https://datadryad.org/resource/doi:10.5061/dryad.8qp4n/2 | | | | | | | |
| Yangon188 | | Yangon, Myanmar | | | | | hspEuropeN | | | | https://figshare.com/articles/Helicobacter_pylori_from_clinical_gastric_infection/5245837 | | | | | | | |
| Yangon179 | | Yangon, Myanmar | | | | | hspAfrica1NAmerica | | | | https://datadryad.org/resource/doi:10.5061/dryad.8qp4n/2 | | | | | | | |
| Yangon173 | | Yangon, Myanmar | | | | | hspAfrica1NAmerica | | | | https://datadryad.org/resource/doi:10.5061/dryad.8qp4n/2 | | | | | | | |
| Yangon159 | | Yangon, Myanmar | | | | | hspAfrica1NAmerica | | | | https://datadryad.org/resource/doi:10.5061/dryad.8qp4n/2 | | | | | | | |
| Yangon142 | | Yangon, Myanmar | | | | | hspEuropeS | | | | https://datadryad.org/resource/doi:10.5061/dryad.8qp4n/2 | | | | | | | |
| **Table S1, Isolate details for the global 565 strains dataset. (page 12)** | | | | | | | | | | | | | | | | |  |  |
| **Isolate** | | **Geographic provenance** | | | | | **FineStructure Population** | | | | **Source** | | | | | | |  |
| Yangon132 | | Yangon, Myanmar | | | | | hspEuropeN | | | | https://figshare.com/articles/Helicobacter_pylori_from_clinical_gastric_infection/5245837 | | | | | | | |
| oki102 | | Okinawa, Japan | | | | | hspAfrica1NAmerica | | | | GCA_000600045.1 | | | | | | | |
| NP05 | | Nepal | | | | | hspAfrica1NAmerica | | | | https://figshare.com/articles/Helicobacter_pylori_from_clinical_gastric_infection/5245837 | | | | | | | |
| NP05-282 | | Nepal | | | | | hspAfrica1NAmerica | | | | https://figshare.com/articles/Helicobacter_pylori_from_clinical_gastric_infection/5245837 | | | | | | | |
| NP05-278 | | Nepal | | | | | hspAfrica1NAmerica | | | | https://datadryad.org/resource/doi:10.5061/dryad.8qp4n/2 | | | | | | | |
| NP05-272 | | Nepal | | | | | hspEuropeN | | | | https://datadryad.org/resource/doi:10.5061/dryad.8qp4n/2 | | | | | | | |
| NP05-266 | | Nepal | | | | | hspAfrica1NAmerica | | | | https://figshare.com/articles/Helicobacter_pylori_from_clinical_gastric_infection/5245837 | | | | | | | |
| NP05-261 | | Nepal | | | | | hspAfrica1NAmerica | | | | https://figshare.com/articles/Helicobacter_pylori_from_clinical_gastric_infection/5245837 | | | | | | | |
| NP05-250 | | Nepal | | | | | hspEuropeN | | | | https://datadryad.org/resource/doi:10.5061/dryad.8qp4n/2 | | | | | | | |
| NP05-234 | | Nepal | | | | | hspAfrica1WAfrica | | | | https://figshare.com/articles/Helicobacter_pylori_from_clinical_gastric_infection/5245837 | | | | | | | |
| NP05-227 | | Nepal | | | | | hspAfrica1NAmerica | | | | https://figshare.com/articles/Helicobacter_pylori_from_clinical_gastric_infection/5245837 | | | | | | | |
| NP05-124 | | Nepal | | | | | hspAfrica1NAmerica | | | | https://figshare.com/articles/Helicobacter_pylori_from_clinical_gastric_infection/5245837 | | | | | | | |
| NP05-121 | | Nepal | | | | | hspAfrica1NAmerica | | | | https://datadryad.org/resource/doi:10.5061/dryad.8qp4n/2 | | | | | | | |
| NP05-112 | | Nepal | | | | | hspAfrica1NAmerica | | | | https://datadryad.org/resource/doi:10.5061/dryad.8qp4n/2 | | | | | | | |
| NP05-107 | | Nepal | | | | | hspAfrica1NAmerica | | | | https://datadryad.org/resource/doi:10.5061/dryad.8qp4n/2 | | | | | | | |
| NP05-105 | | Nepal | | | | | hspEuropeN | | | | https://datadryad.org/resource/doi:10.5061/dryad.8qp4n/2 | | | | | | | |
| NP04 | | Nepal | | | | | hspEuropeN | | | | https://datadryad.org/resource/doi:10.5061/dryad.8qp4n/2 | | | | | | | |
| Myanmar66 | | Myanmar | | | | | hspAfrica1NAmerica | | | | https://datadryad.org/resource/doi:10.5061/dryad.8qp4n/2 | | | | | | | |
| Myanmar52 | | Myanmar | | | | | hspAfrica1NAmerica | | | | https://datadryad.org/resource/doi:10.5061/dryad.8qp4n/2 | | | | | | | |
| Myanmar51 | | Myanmar | | | | | hspAfrica1NAmerica | | | | https://datadryad.org/resource/doi:10.5061/dryad.8qp4n/2 | | | | | | | |
| Mandalay60 | | Mandalay, Myanmar | | | | | hspAfrica1NAmerica | | | | https://figshare.com/articles/Helicobacter_pylori_from_clinical_gastric_infection/5245837 | | | | | | | |
| Mandalay46 | | Mandalay, Myanmar | | | | | hspAfrica1NAmerica | | | | https://figshare.com/articles/Helicobacter_pylori_from_clinical_gastric_infection/5245837 | | | | | | | |
| Mandalay38 | | Mandalay, Myanmar | | | | | hspAfrica1NAmerica | | | | https://datadryad.org/resource/doi:10.5061/dryad.8qp4n/2 | | | | | | | |
| Mandalay30 | | Mandalay, Myanmar | | | | | hspAfrica1NAmerica | | | | https://datadryad.org/resource/doi:10.5061/dryad.8qp4n/2 | | | | | | | |
| Mandalay13 | | Mandalay, Myanmar | | | | | hspAfrica1NAmerica | | | | https://figshare.com/articles/Helicobacter_pylori_from_clinical_gastric_infection/5245837 | | | | | | | |
| Mandalay03 | | Mandalay, Myanmar | | | | | hspEuropeS | | | | https://datadryad.org/resource/doi:10.5061/dryad.8qp4n/2 | | | | | | | |
| oki112 | | Okinawa, Japan | | | | | hspAfrica1NAmerica | | | | GCA_000600085.1 | | | | | | | |
| oki128 | | Okinawa, Japan | | | | | hspEuropeN | | | | GCA_000600125.1 | | | | | | | |
| oki154 | | Okinawa, Japan | | | | | hspAfrica1NAmerica | | | | GCA_000600145.1 | | | | | | | |
| oki673 | | Okinawa, Japan | | | | | hspAfrica1NAmerica | | | | GCA_000600185.1 | | | | | | | |
| oki828 | | Okinawa, Japan | | | | | hspEuropeN | | | | GCA_000600205.1 | | | | | | | |
| **Table S1, Isolate details for the global 565 strains dataset. (page 13)** | | | | | | | | | | | | | | | | |  |  |
| **Isolate** | | **Geographic provenance** | | | | | **FineStructure Population** | | | | **Source** | | | | | | |  |
| oki898 | | Okinawa, Japan | | | | | hspAfrica1NAmerica | | | | GCA_000600225.1 | | | | | | | |
| J166 | | Nashville, Tenessee | | | | | hspAfrica1NAmerica | | | | GCA_000685625.1 | | | | | | | |
| BM013A | | Perth, Australia | | | | | hspAfrica1NAmerica | | | | GCA_000685665.1 | | | | | | | |
| Hp238 | | Taiwan | | | | | hspEuropeN | | | | GCA_000817025.1 | | | | | | | |
| BM012A | | Perth, Australia | | | | | hspEuropeN | | | | GCA_000498315.1 | | | | | | | |
| Nic01_A | | Nicaragua | | | | | hspAfrica1NAmerica | | | | https://datadryad.org/resource/doi:10.5061/dryad.8qp4n/2 | | | | | | | |
| Nic03_A | | Nicaragua | | | | | hspEuropeN | | | | https://datadryad.org/resource/doi:10.5061/dryad.8qp4n/2 | | | | | | | |
| Nic04_A | | Nicaragua | | | | | hspAfrica1WAfrica | | | | https://datadryad.org/resource/doi:10.5061/dryad.8qp4n/2 | | | | | | | |
| Nic05_A | | Nicaragua | | | | | hspAfrica1NAmerica | | | | https://datadryad.org/resource/doi:10.5061/dryad.8qp4n/2 | | | | | | | |
| Nic06_A | | Nicaragua | | | | | hspAfrica1NAmerica | | | | https://datadryad.org/resource/doi:10.5061/dryad.8qp4n/2 | | | | | | | |
| Nic07_A | | Nicaragua | | | | | hspAfrica1NAmerica | | | | https://datadryad.org/resource/doi:10.5061/dryad.8qp4n/2 | | | | | | | |
| Nic07_C | | Nicaragua | | | | | hspEuropeN | | | | https://figshare.com/articles/Helicobacter_pylori_from_clinical_gastric_infection/5245837 | | | | | | | |
| Nic08_C2 | | Nicaragua | | | | | hspAfrica1NAmerica | | | | https://datadryad.org/resource/doi:10.5061/dryad.8qp4n/2 | | | | | | | |
| Nic09_A | | Nicaragua | | | | | hspAfrica1WAfrica | | | | https://datadryad.org/resource/doi:10.5061/dryad.8qp4n/2 | | | | | | | |
| Nic10_A | | Nicaragua | | | | | hspAfrica1NAmerica | | | | https://datadryad.org/resource/doi:10.5061/dryad.8qp4n/2 | | | | | | | |
| Nic11_A | | Nicaragua | | | | | hspEuropeN | | | | https://datadryad.org/resource/doi:10.5061/dryad.8qp4n/2 | | | | | | | |
| Nic12_A | | Nicaragua | | | | | hspAfrica1NAmerica | | | | https://datadryad.org/resource/doi:10.5061/dryad.8qp4n/2 | | | | | | | |
| Nic12_C | | Nicaragua | | | | | hspEuropeN | | | | https://figshare.com/articles/Helicobacter_pylori_from_clinical_gastric_infection/5245837 | | | | | | | |
| Nic13_A | | Nicaragua | | | | | hpAsia2 | | | | https://datadryad.org/resource/doi:10.5061/dryad.8qp4n/2 | | | | | | | |
| Nic14_A | | Nicaragua | | | | | hspEuropeN | | | | https://datadryad.org/resource/doi:10.5061/dryad.8qp4n/2 | | | | | | | |
| Nic14_C | | Nicaragua | | | | | hspEuropeN | | | | https://datadryad.org/resource/doi:10.5061/dryad.8qp4n/2 | | | | | | | |
| Nic15_A | | Nicaragua | | | | | hspEuropeColombia | | | | https://datadryad.org/resource/doi:10.5061/dryad.8qp4n/2 | | | | | | | |
| Nic16_A | | Nicaragua | | | | | hspEuropeColombia | | | | https://datadryad.org/resource/doi:10.5061/dryad.8qp4n/2 | | | | | | | |
| Nic17_A | | Nicaragua | | | | | hspEuropeN | | | | https://datadryad.org/resource/doi:10.5061/dryad.8qp4n/2 | | | | | | | |
| Nic18_A | | Nicaragua | | | | | hspEuropeN | | | | https://datadryad.org/resource/doi:10.5061/dryad.8qp4n/2 | | | | | | | |
| Nic19_A | | Nicaragua | | | | | hspEuropeN | | | | https://datadryad.org/resource/doi:10.5061/dryad.8qp4n/2 | | | | | | | |
| Nic19_C | | Nicaragua | | | | | hspEuropeN | | | | https://figshare.com/articles/Helicobacter_pylori_from_clinical_gastric_infection/5245837 | | | | | | | |
| Nic20_A | | Nicaragua | | | | | hspEuropeN | | | | https://datadryad.org/resource/doi:10.5061/dryad.8qp4n/2 | | | | | | | |
| Nic20_C | | Nicaragua | | | | | hspEuropeS | | | | https://datadryad.org/resource/doi:10.5061/dryad.8qp4n/2 | | | | | | | |
| Nic21_C | | Nicaragua | | | | | hspEuropeN | | | | https://datadryad.org/resource/doi:10.5061/dryad.8qp4n/2 | | | | | | | |
| NQ315 | | Narino, Colombia | | | | | hspEuropeN | | | | GCA_000285075.1 | | | | | | | |
| **Table S1, Isolate details for the global 565 strains dataset. (page 14)** | | | | | | | | | | | | | | | | |  |  |
| **Isolate** | | **Geographic provenance** | | | | | **FineStructure Population** | | | | **Source** | | | | | | |  |
| NQ392 | | Narino, Colombia | | | | | hspEuropeN | | | | GCA_000285155.1 | | | | | | | |
| YN1-91 | | Kunming, Yunnan, China | | | | | hspAfrica1NAmerica | | | | GCA_000740095.1 | | | | | | | |
| YN4-84 | | Lijiang, Yunnan, China | | | | | hspEuropeN | | | | GCA_000740115.1 | | | | | | | |
| Manado-1 | | North Sulawesi, Indonesia | | | | | hspEuropeN | | | | GCA_000766035.1 | | | | | | | |
| Taiwan-47 | | Taiwan | | | | | hspEuropeN | | | | GCA_000766025.1 | | | | | | | |
| CA2_A | | Nicaragua | | | | | hspEuropeN | | | | https://figshare.com/articles/Helicobacter_pylori_from_clinical_gastric_infection/5245837 | | | | | | | |
| Nic_2A | | Nicaragua | | | | | hspEuropeN | | | | https://datadryad.org/resource/doi:10.5061/dryad.8qp4n/2 | | | | | | | |
| Nic_2B | | Nicaragua | | | | | hspEuropeN | | | | https://datadryad.org/resource/doi:10.5061/dryad.8qp4n/2 | | | | | | | |
| Nic_2C | | Nicaragua | | | | | hspEuropeN | | | | https://datadryad.org/resource/doi:10.5061/dryad.8qp4n/2 | | | | | | | |
| Nic_2D | | Nicaragua | | | | | hspEuropeColombia | | | | https://datadryad.org/resource/doi:10.5061/dryad.8qp4n/2 | | | | | | | |
| Nic_B | | Nicaragua | | | | | hspEuropeN | | | | https://datadryad.org/resource/doi:10.5061/dryad.8qp4n/2 | | | | | | | |
| Nic_D | | Nicaragua | | | | | hspEuropeN | | | | https://datadryad.org/resource/doi:10.5061/dryad.8qp4n/2 | | | | | | | |
| Nic_F | | Nicaragua | | | | | hspEuropeN | | | | https://datadryad.org/resource/doi:10.5061/dryad.8qp4n/2 | | | | | | | |
| Nic_G | | Nicaragua | | | | | hspEuropeN | | | | https://datadryad.org/resource/doi:10.5061/dryad.8qp4n/2 | | | | | | | |
| Nic_H | | Nicaragua | | | | | hspEuropeN | | | | https://datadryad.org/resource/doi:10.5061/dryad.8qp4n/2 | | | | | | | |
| 21580 | | Belgium | | | | | hspEuropeN | | | | SAMN08381241 | | | | | | | |
| 30908 | | Belgium | | | | | hspEuropeN | | | | SAMN08381242 | | | | | | | |
| 30950 | | Belgium | | | | | hspEuropeN | | | | SAMN08381243 | | | | | | | |
| 31235 | | Belgium | | | | | hspEuropeN | | | | SAMN08381244 | | | | | | | |
| 36166 | | Belgium | | | | | hspEuropeN | | | | SAMN08381245 | | | | | | | |
| 38185 | | Belgium | | | | | hspEuropeColombia | | | | SAMN08381246 | | | | | | | |
| 444 | | Nottingham, UK | | | | | hspEuropeN | | | | SAMN08381247 | | | | | | | |
| 448 | | Nottingham, UK | | | | | hspEuropeN | | | | SAMN08381248 | | | | | | | |
| 456 | | Nottingham, UK | | | | | hspEuropeN | | | | SAMN08381249 | | | | | | | |
| 462 | | Nottingham, UK | | | | | hspEuropeN | | | | SAMN08381250 | | | | | | | |
| 518 | | Nottingham, UK | | | | | hspEuropeN | | | | SAMN08381251 | | | | | | | |
| 638 | | Nottingham, UK | | | | | hspEuropeN | | | | SAMN08381252 | | | | | | | |
| HE_C1 | | Sweden | | | | | hspEuropeN | | | | SAMN08381253 | | | | | | | |
| HE_C10 | | Sweden | | | | | hspEuropeN | | | | SAMN08381254 | | | | | | | |
| HE_C11 | | Sweden | | | | | hspEuropeN | | | | SAMN08381255 | | | | | | | |
| HE_C12 | | Sweden | | | | | hspEuropeN | | | | SAMN08381256 | | | | | | | |
| **Table S1, Isolate details for the global 565 strains dataset. (page 15)** | | | | | | | | | | | | | | | | |  |  |
| **Isolate** | | **Geographic provenance** | | | | | **FineStructure Population** | | | | **Source** | | | | | | |  |
| HE_C13 | | Sweden | | | | | hspEuropeN | | | | SAMN08381257 | | | | | | | |
| HE_C14 | | Sweden | | | | | hspEuropeN | | | | SAMN08381258 | | | | | | | |
| HE_C15 | | Sweden | | | | | hspEuropeN | | | | SAMN08381259 | | | | | | | |
| HE_C16 | | Sweden | | | | | hspEuropeN | | | | SAMN08381260 | | | | | | | |
| HE_C17 | | Sweden | | | | | hspEuropeN | | | | SAMN08381261 | | | | | | | |
| HE_C18 | | Sweden | | | | | hspEuropeN | | | | SAMN08381262 | | | | | | | |
| HE_C19 | | Sweden | | | | | hspEuropeN | | | | SAMN08381263 | | | | | | | |
| HE_C20 | | Sweden | | | | | hspEuropeN | | | | SAMN08381264 | | | | | | | |
| HE_C21 | | Sweden | | | | | hspEuropeN | | | | SAMN08381265 | | | | | | | |
| HE_C23 | | Sweden | | | | | hspEuropeN | | | | SAMN08381266 | | | | | | | |
| HE_C3 | | Sweden | | | | | hspEuropeColombia | | | | SAMN08381267 | | | | | | | |
| HE_C4 | | Sweden | | | | | hspEuropeN | | | | SAMN08381268 | | | | | | | |
| HE_C5 | | Sweden | | | | | hspEuropeN | | | | SAMN08381269 | | | | | | | |
| HE_C7 | | Sweden | | | | | hspEuropeN | | | | SAMN08381270 | | | | | | | |
| HE_C8 | | Sweden | | | | | hspEuropeN | | | | SAMN08381271 | | | | | | | |
| HE_C9 | | Sweden | | | | | hspEuropeN | | | | SAMN08381272 | | | | | | | |
| HE_NC1 | | Sweden | | | | | hspEuropeN | | | | SAMN08381273 | | | | | | | |
| HE_NC11 | | Sweden | | | | | hspEuropeN | | | | SAMN08381274 | | | | | | | |
| HE_NC12 | | Sweden | | | | | hspEuropeN | | | | SAMN08381275 | | | | | | | |
| HE_NC13 | | Sweden | | | | | hspEuropeColombia | | | | SAMN08381276 | | | | | | | |
| HE_NC14 | | Sweden | | | | | hspEuropeN | | | | SAMN08381277 | | | | | | | |
| HE_NC15 | | Sweden | | | | | hspEuropeN | | | | SAMN08381278 | | | | | | | |
| HE_NC16 | | Sweden | | | | | hspEuropeN | | | | SAMN08381279 | | | | | | | |
| HE_NC17 | | Sweden | | | | | hspEuropeN | | | | SAMN08381280 | | | | | | | |
| HE_NC18 | | Sweden | | | | | hspEuropeN | | | | SAMN08381281 | | | | | | | |
| HE_NC19 | | Sweden | | | | | hspEuropeN | | | | SAMN08381282 | | | | | | | |
| HE_NC2 | | Sweden | | | | | hspEuropeN | | | | SAMN08381283 | | | | | | | |
| HE_NC20 | | Sweden | | | | | hspEuropeN | | | | SAMN08381284 | | | | | | | |
| HE_NC21 | | Sweden | | | | | hspEuropeN | | | | SAMN08381285 | | | | | | | |
| HE_NC22 | | Sweden | | | | | hspEuropeN | | | | SAMN08381286 | | | | | | | |
| HE_NC23 | | Sweden | | | | | hspEuropeS | | | | SAMN08381287 | | | | | | | |
| **Table S1, Isolate details for the global 565 strains dataset. (page 16)** | | | | | | | | | | | | | | | | |  |  |
| **Isolate** | | **Geographic provenance** | | | | | **FineStructure Population** | | | | **Source** | | | | | | |  |
| HE_NC24 | | Sweden | | | | | hspEuropeN | | | | SAMN08381288 | | | | | | | |
| HE_NC25 | | Sweden | | | | | hspEuropeN | | | | SAMN08381289 | | | | | | | |
| HE_NC26 | | Sweden | | | | | hspEuropeN | | | | SAMN08381290 | | | | | | | |
| HE_NC28 | | Sweden | | | | | hspEuropeColombia | | | | SAMN08381291 | | | | | | | |
| HE_NC29 | | Sweden | | | | | hspEuropeS | | | | SAMN08381292 | | | | | | | |
| HE_NC3 | | Sweden | | | | | hspEuropeColombia | | | | SAMN08381293 | | | | | | | |
| HE_NC30 | | Sweden | | | | | hspAfrica1Americas | | | | SAMN08381294 | | | | | | | |
| HE_NC32 | | Sweden | | | | | hspEuropeS | | | | SAMN08381295 | | | | | | | |
| HE_NC33 | | Sweden | | | | | hspEuropeColombia | | | | SAMN08381296 | | | | | | | |
| HE_NC34 | | Sweden | | | | | hspEuropeColombia | | | | SAMN08381297 | | | | | | | |
| HE_NC35 | | Sweden | | | | | hspAfrica1Americas | | | | SAMN08381298 | | | | | | | |
| HE_NC36 | | Sweden | | | | | hspEuropeColombia | | | | SAMN08381299 | | | | | | | |
| HE_NC38 | | Sweden | | | | | hspEuropeS | | | | SAMN08381300 | | | | | | | |
| HE_NC39 | | Sweden | | | | | hspEuropeS | | | | SAMN08381301 | | | | | | | |
| HE_NC40 | | Sweden | | | | | hspEuropeS | | | | SAMN08381302 | | | | | | | |
| HE_NC41 | | Sweden | | | | | hspAfrica1Americas | | | | SAMN08381303 | | | | | | | |
| HE_NC42 | | Sweden | | | | | hspEuropeColombia | | | | SAMN08381304 | | | | | | | |
| HE_NC43 | | Sweden | | | | | hspEuropeS | | | | SAMN08381305 | | | | | | | |
| HE_NC54 | | Sweden | | | | | hspEuropeS | | | | SAMN08381306 | | | | | | | |
| HE_NC6 | | Sweden | | | | | hspAfrica1Americas | | | | SAMN08381307 | | | | | | | |
| HE_NC7 | | Sweden | | | | | hspEuropeColombia | | | | SAMN08381308 | | | | | | | |
| 2012-26 | | Mexico | | | | | hspEuropeS | | | | GCA_002026865.1 | | | | | | | |
| 22025 | | Colombia | | | | | hspAfrica1Americas | | | | GCA_002012725.1 | | | | | | | |
| ms167 | | Mexico | | | | | hspEuropeColombia | | | | GCA_002026705.1 | | | | | | | |
| ms1055 | | Mexico | | | | | hspEuropeS | | | | GCA_002026885.1 | | | | | | | |
| 22402 | | Colombia | | | | | hspEuropeColombia | | | | GCA_002012655.1 | | | | | | | |
| 22087_ve | | Colombia | | | | | hspEuropeS | | | | GCA_002012795.1 | | | | | | | |
| ms1063 | | Mexico | | | | | hspEuropeS | | | | GCA_002026895.1 | | | | | | | |
| 26084 | | Colombia | | | | | hspEuropeColombia | | | | GCA_002012685.1 | | | | | | | |
| 2004-20 | | Mexico | | | | | hspEuropeColombia | | | | GCA_002026785.1 | | | | | | | |
| 2005-98 | | Mexico | | | | | hspEuropeS | | | | GCA_002026595.1 | | | | | | | |
| **Table S1, Isolate details for the global 565 strains dataset. (page 17)** | | | | | | | | | | | | | | | | |  |  |
| **Isolate** | | **Geographic provenance** | | | | | **FineStructure Population** | | | | **Source** | | | | | | |  |
| ms203 | | Mexico | | | | | hspEuropeColombia | | | | GCA_002026955.1 | | | | | | | |
| 2005-72 | | Mexico | | | | | hspEuropeColombia | | | | GCA_002027065.1 | | | | | | | |
| 26093 | | Colombia | | | | | hspEuropeS | | | | GCA_002012745.1 | | | | | | | |
| ms1078 | | Mexico | | | | | hspEuropeS | | | | GCA_002027025.1 | | | | | | | |
| 2006-52 | | Mexico | | | | | hspEuropeS | | | | GCA_002026835.1 | | | | | | | |
| 2003-98 | | Mexico | | | | | hspEuropeS | | | | GCA_002026545.1 | | | | | | | |
| 2006-407 | | Mexico | | | | | hspEuropeColombia | | | | GCA_002026555.1 | | | | | | | |
| 2006-103 | | Mexico | | | | | hspEuropeS | | | | GCA_002026805.1 | | | | | | | |
| 22346 | | Colombia | | | | | hspEuropeColombia | | | | GCA_002013065.1 | | | | | | | |
| ms15 | | Mexico | | | | | hspEuropeColombia | | | | GCA_002026675.1 | | | | | | | |
| 22337 | | Colombia | | | | | hspEuropeS | | | | GCA_002012495.1 | | | | | | | |
| ms23 | | Mexico | | | | | hspAfrica1Americas | | | | GCA_002026755.1 | | | | | | | |
| ms2_ve | | Mexico | | | | | hspAfrica1Americas | | | | GCA_002026745.1 | | | | | | | |
| 22341_ve | | Colombia | | | | | hspEuropeColombia | | | | GCA_002013055.1 | | | | | | | |
| 2006-56 | | Mexico | | | | | hspAfrica1Americas | | | | GCA_002027125.1 | | | | | | | |
| 22023 | | Colombia | | | | | hspEuropeColombia | | | | GCA_002012715.1 | | | | | | | |
| 22327 | | Colombia | | | | | hspEuropeColombia | | | | GCA_002012475.1 | | | | | | | |
| ms931 | | Mexico | | | | | hspEuropeS | | | | GCA_002026455.1 | | | | | | | |
| 2005-100 | | Mexico | | | | | hspEuropeS | | | | GCA_002026485.1 | | | | | | | |
| ms1080 | | Mexico | | | | | hspEuropeColombia | | | | GCA_002026925.1 | | | | | | | |
| ms13 | | Mexico | | | | | hspEuropeColombia | | | | GCA_002026665.1 | | | | | | | |
| 26100 | | Colombia | | | | | hspAfrica1Americas | | | | GCA_002012755.1 | | | | | | | |
| 2003-84 | | Mexico | | | | | hspEuropeS | | | | GCA_002027045.1 | | | | | | | |
| 22046_ve | | Colombia | | | | | hspEuropeColombia | | | | GCA_002013035.1 | | | | | | | |
| 2006-479 | | Mexico | | | | | hspAfrica1Americas | | | | GCA_002026645.1 | | | | | | | |
| ms1054 | | Mexico | | | | | hspEuropeN | | | | GCA_002026505.1 | | | | | | | |
| ms44 | | Mexico | | | | | hspEuropeS | | | | GCA_002026985.1 | | | | | | | |
| 22389 | | Colombia | | | | | hspEuropeColombia | | | | GCA_002012935.1 | | | | | | | |
| 22366_ve | | Colombia | | | | | hspEuropeColombia | | | | GCA_002012855.1 | | | | | | | |
| 22013 | | Colombia | | | | | hspEuropeColombia | | | | GCA_002012945.1 | | | | | | | |
| 22362 | | Colombia | | | | | hspEuropeColombia | | | | GCA_002012565.1 | | | | | | | |
| **Table S1, Isolate details for the global 565 strains dataset. (page 18)** | | | | | | | | | | | | | | | | |  |  |
| **Isolate** | | **Geographic provenance** | | | | | **FineStructure Population** | | | | **Source** | | | | | | |  |
| 2005-126 | | Mexico | | | | | hspAfrica1Americas | | | | GCA_002027005.1 | | | | | | | |
| 2003-103 | | Mexico | | | | | hspEuropeColombia | | | | GCA_002026445.1 | | | | | | | |
| 22367_ve | | Colombia | | | | | hspEuropeColombia | | | | GCA_002012815.1 | | | | | | | |
| 22021 | | Colombia | | | | | hspEuropeS | | | | GCA_002012965.1 | | | | | | | |
| 2003-107 | | Mexico | | | | | hspAfrica1SAfrica | | | | GCA_002026935.1 | | | | | | | |
| 2006-480 | | Mexico | | | | | hspEuropeColombia | | | | GCA_002027085.1 | | | | | | | |
| 22385 | | Colombia | | | | | hspEuropeColombia | | | | GCA_002012955.1 | | | | | | | |
| 24004 | | Colombia | | | | | hspEuropeColombia | | | | GCA_002012575.1 | | | | | | | |
| 2006-4 | | Mexico | | | | | hspEuropeColombia | | | | GCA_002026625.1 | | | | | | | |
| 22370 | | Colombia | | | | | hspEuropeColombia | | | | GCA_002012915.1 | | | | | | | |
| 22311 | | Colombia | | | | | hspEuropeColombia | | | | GCA_002012505.1 | | | | | | | |
| 22390 | | Colombia | | | | | hspAfrica1Americas | | | | GCA_002013205.1 | | | | | | | |
| 22339 | | Colombia | | | | | hspEuropeColombia | | | | GCA_002012555.1 | | | | | | | |
| 2011-145 | | Mexico | | | | | hspEuropeN | | | | GCA_002026515.1 | | | | | | | |
| 22312 | | Colombia | | | | | hspEuropeColombia | | | | GCA_002012395.1 | | | | | | | |
| 22322 | | Colombia | | | | | hspEuropeColombia | | | | GCA_002012805.1 | | | | | | | |
| 2004-2 | | Mexico | | | | | hspEuropeS | | | | GCA_002026825.1 | | | | | | | |
| 22331 | | Colombia | | | | | hspEuropeColombia | | | | GCA_002013045.1 | | | | | | | |
| 26024 | | Colombia | | | | | hspEuropeColombia | | | | GCA_002012635.1 | | | | | | | |
| 22368 | | Colombia | | | | | hspEuropeColombia | | | | GCA_002013175.1 | | | | | | | |
| 22360 | | Colombia | | | | | hspEuropeColombia | | | | GCA_002013145.1 | | | | | | | |
| 22378 | | Colombia | | | | | hspEuropeN | | | | GCA_002012885.1 | | | | | | | |
| 22019_ve | | Colombia | | | | | hspEuropeS | | | | GCA_002012405.1 | | | | | | | |
| 22371 | | Colombia | | | | | hspEuropeColombia | | | | GCA_002012875.1 | | | | | | | |
| 22020 | | Colombia | | | | | hspEuropeColombia | | | | GCA_002012415.1 | | | | | | | |
| 22315_ve | | Colombia | | | | | hspEuropeColombia | | | | GCA_002013015.1 | | | | | | | |
| 22335 | | Colombia | | | | | hspEuropeColombia | | | | GCA_002012485.1 | | | | | | | |
| ms176 | | Mexico | | | | | hspEuropeN | | | | GCA_002026725.1 | | | | | | | |
| 22393 | | Colombia | | | | | hspEuropeS | | | | GCA_002012615.1 | | | | | | | |
| 22093 | | Colombia | | | | | hspEuropeN | | | | GCA_002012645.1 | | | | | | | |
| 22095 | | Colombia | | | | | hspEuropeN | | | | GCA_002012425.1 | | | | | | | |
| **Table S1, Isolate details for the global 565 strains dataset. (page 19)** | | | | | | | | | | | | | | | | |  |  |
| **Isolate** | | **Geographic provenance** | | | | | **FineStructure Population** | | | | **Source** | | | | | | |  |
| 22347 | | Colombia | | | | | hspEuropeN | | | | GCA_002013115.1 | | | | | | | |
| 2011-41 | | Mexico | | | | | hspEuropeS | | | | GCA_002026585.1 | | | | | | | |
| 22388 | | Colombia | | | | | hspEAsia | | | | GCA_002013185.1 | | | | | | | |
| 22351 | | Colombia | | | | | hpAsia2 | | | | GCA_002013135.1 | | | | | | | |
| 22384 | | Colombia | | | | | hspEAsia | | | | https://datadryad.org/resource/doi:10.5061/dryad.8qp4n/2 | | | | | | | |
| 24008 | | Colombia | | | | | hspEuropeN | | | | https://datadryad.org/resource/doi:10.5061/dryad.8qp4n/2 | | | | | | | |
| ms965_ve | | Mexico | | | | | hpAsia2 | | | | https://datadryad.org/resource/doi:10.5061/dryad.8qp4n/2 | | | | | | | |
|  |  | | | | | |  | | | |  | | | | | | |  |
|  | | | | | | | | | | |  | | | | | | |  |
|  | | | | | | | | | | | | | | | | | |  |
| **Table S2, Isolate details for the hpEurope GWAS dataset. (page 1)** | | | | | | | | | | | | | | | | | |  |
| **Isolate** | **Host Pathology** | | | | | | | | | **GWAS group** | | | | | | **Isolation Country** | |  |
| 26695_Tomb | Gastritis | | | | | | | | | Non Atrophic Gastritis | | | | | | UK | |  |
| ELS37 | gastric cancer | | | | | | | | | Gastric Cancer | | | | | | El Salvador | |  |
| HPAG1 | atrophic gastritis | | | | | | | | | Progressive towards Cancer | | | | | | Sweden | |  |
| SJM180 | Gastritis | | | | | | | | | Non Atrophic Gastritis | | | | | | Peru | |  |
| Hp_A-14 | Gastritis | | | | | | | | | Non Atrophic Gastritis | | | | | | USA | |  |
| Hp_A-26 | Gastritis | | | | | | | | | Non Atrophic Gastritis | | | | | | USA | |  |
| Hp_A-27 | Gastritis | | | | | | | | | Non Atrophic Gastritis | | | | | | USA | |  |
| Hp_H-11 | Gastritis | | | | | | | | | Non Atrophic Gastritis | | | | | | USA | |  |
| Hp_H-9 | Gastritis | | | | | | | | | Non Atrophic Gastritis | | | | | | USA | |  |
| Hp_P-15 | Gastritis | | | | | | | | | Non Atrophic Gastritis | | | | | | USA | |  |
| Hp_P-16 | Gastritis | | | | | | | | | Non Atrophic Gastritis | | | | | | USA | |  |
| Hp_P-23 | Gastritis | | | | | | | | | Non Atrophic Gastritis | | | | | | USA | |  |
| Hp_P-30 | Gastritis | | | | | | | | | Non Atrophic Gastritis | | | | | | USA | |  |
| Hp_P-74 | Gastritis | | | | | | | | | Non Atrophic Gastritis | | | | | | USA | |  |
| N6 | Gastritis | | | | | | | | | Non Atrophic Gastritis | | | | | | Burma | |  |
| NQ4200 | intestinal metaplasia | | | | | | | | | Progressive towards Cancer | | | | | | Colombia | |  |
| NQ4228 | intestinal metaplasia | | | | | | | | | Progressive towards Cancer | | | | | | Colombia | |  |
| R037c | asymptomatic | | | | | | | | | Non Atrophic Gastritis | | | | | | Canada | |  |
| R038b | asymptomatic | | | | | | | | | Non Atrophic Gastritis | | | | | | Canada | |  |
| R046Wa | asymptomatic | | | | | | | | | Non Atrophic Gastritis | | | | | | Canada | |  |
| R32b | asymptomatic | | | | | | | | | Non Atrophic Gastritis | | | | | | Canada | |  |
| UM037 | Stomach fundus tumor | | | | | | | | | Gastric Cancer | | | | | | Malaysia | |  |
| GC11-HL | Non cardia gastric cancer instestinal type | | | | | | | | | Gastric Cancer | | | | | | France | |  |
| GC23-HL | Non cardia gastric cancer IT | | | | | | | | | Gastric Cancer | | | | | | France | |  |
| GC26-HL | Non cardia gastric cancer DT | | | | | | | | | Gastric Cancer | | | | | | France | |  |
| GC27-HL | Non cardia gastric cancer IT | | | | | | | | | Gastric Cancer | | | | | | France | |  |
| GC31-B | GIST | | | | | | | | | Gastric Cancer | | | | | | France | |  |
| GC34-HL | gastric cancer | | | | | | | | | Gastric Cancer | | | | | | France | |  |
| GC43-HL | Non cardia gastric cancer IT | | | | | | | | | Gastric Cancer | | | | | | France | |  |
| GC54-HL | Non cardia gastric cancer IT | | | | | | | | | Gastric Cancer | | | | | | France | |  |
| GC65-HL | gastric cancer | | | | | | | | | Gastric Cancer | | | | | | France | |  |
| GC67-HL | gastric cancer | | | | | | | | | Gastric Cancer | | | | | | France | |  |
| GC30-HL | Non cardia gastric cancer DT | | | | | | | | | Gastric Cancer | | | | | | France | |  |
| GC52-HL | Non cardia gastric cancer IT | | | | | | | | | Gastric Cancer | | | | | | France | |  |
| **Table S2, Isolate details for the hpEurope GWAS dataset. (page 2)** | | | | | | | | | | | | | | | | | |  |
| **Isolate** | **Host Pathology** | | | | | | | | | **GWAS group** | | | | | | **Isolation Country** | |  |
| 3800 | Gastritis | | | | | | | | | Non Atrophic Gastritis | | | | | | France | |  |
| 3745 | Gastritis | | | | | | | | | Non Atrophic Gastritis | | | | | | France | |  |
| 3697 | Gastritis | | | | | | | | | Non Atrophic Gastritis | | | | | | France | |  |
| 3699 | Gastritis | | | | | | | | | Non Atrophic Gastritis | | | | | | France | |  |
| 3746 | Gastritis | | | | | | | | | Non Atrophic Gastritis | | | | | | France | |  |
| GC69-HL | gastric cancer | | | | | | | | | Gastric Cancer | | | | | | France | |  |
| BM013A | Asymptomatic | | | | | | | | | Non Atrophic Gastritis | | | | | | Australia | |  |
| BM012A | Asymptomatic | | | | | | | | | Non Atrophic Gastritis | | | | | | Australia | |  |
| Nic20_A | Intestinal metaplasia and atrophy | | | | | | | | | Progressive towards Cancer | | | | | | Nicaragua | |  |
| 21580 | gastric cancer | | | | | | | | | Gastric Cancer | | | | | | Belgium | |  |
| 30908 | Normal stomach | | | | | | | | | Non Atrophic Gastritis | | | | | | Belgium | |  |
| 30950 | gastric cancer | | | | | | | | | Gastric Cancer | | | | | | Belgium | |  |
| 31235 | Normal stomach | | | | | | | | | Non Atrophic Gastritis | | | | | | Belgium | |  |
| 36166 | Normal stomach | | | | | | | | | Non Atrophic Gastritis | | | | | | Belgium | |  |
| 38185 | gastric cancer | | | | | | | | | Gastric Cancer | | | | | | Belgium | |  |
| 448 | Normal stomach | | | | | | | | | Non Atrophic Gastritis | | | | | | UK | |  |
| 456 | Normal stomach | | | | | | | | | Non Atrophic Gastritis | | | | | | UK | |  |
| 518 | Normal stomach | | | | | | | | | Non Atrophic Gastritis | | | | | | UK | |  |
| HE_C1 | Non-cardia gastric cancer | | | | | | | | | Gastric Cancer | | | | | | Sweden | |  |
| HE_C32 | Non-cardia gastric cancer | | | | | | | | | Gastric Cancer | | | | | | Sweden | |  |
| HE_C33 | Non-cardia gastric cancer | | | | | | | | | Gastric Cancer | | | | | | Sweden | |  |
| HE_C34 | Non-cardia gastric cancer | | | | | | | | | Gastric Cancer | | | | | | Sweden | |  |
| HE_C38 | Non-cardia gastric cancer | | | | | | | | | Gastric Cancer | | | | | | Sweden | |  |
| HE_C40 | Non-cardia gastric cancer | | | | | | | | | Gastric Cancer | | | | | | Sweden | |  |
| HE_C50 | Non-cardia gastric cancer | | | | | | | | | Gastric Cancer | | | | | | Sweden | |  |
| HE_C52 | Non-cardia gastric cancer | | | | | | | | | Gastric Cancer | | | | | | Sweden | |  |
| HE_C55 | Non-cardia gastric cancer | | | | | | | | | Gastric Cancer | | | | | | Sweden | |  |
| HE_C57 | Cardia gastric cancer | | | | | | | | | Gastric Cancer | | | | | | Sweden | |  |
| HE_C58 | Non-cardia gastric cancer | | | | | | | | | Gastric Cancer | | | | | | Sweden | |  |
| HE_C59 | Non-cardia gastric cancer | | | | | | | | | Gastric Cancer | | | | | | Sweden | |  |
| HE_C66 | Non-cardia gastric cancer | | | | | | | | | Gastric Cancer | | | | | | Sweden | |  |
| HE_C73 | Non-cardia gastric cancer | | | | | | | | | Gastric Cancer | | | | | | Sweden | |  |
| HE_C9 | Non-cardia gastric cancer | | | | | | | | | Gastric Cancer | | | | | | Sweden | |  |
| HE_C11 | Non-cardia gastric cancer | | | | | | | | | Gastric Cancer | | | | | | Sweden | |  |
| **Table S2, Isolate details for the hpEurope GWAS dataset. (page 3)** | | | | | | | | | | | | | | | | | |  |
| **Isolate** | **Host Pathology** | | | | | | | | | **GWAS group** | | | | | | **Isolation Country** | |  |
| HE_C13 | Non-cardia gastric cancer | | | | | | | | | Gastric Cancer | | | | | | Sweden | |  |
| HE_C18 | Non-cardia gastric cancer | | | | | | | | | Gastric Cancer | | | | | | Sweden | |  |
| HE_C23 | Non-cardia gastric cancer | | | | | | | | | Gastric Cancer | | | | | | Sweden | |  |
| HE_C30 | Non-cardia gastric cancer | | | | | | | | | Gastric Cancer | | | | | | Sweden | |  |
| HE_NC1-1 | Non-atrophic gastritis | | | | | | | | | Non Atrophic Gastritis | | | | | | Sweden | |  |
| HE_NC13-6 | Non-atrophic gastritis | | | | | | | | | Non Atrophic Gastritis | | | | | | Sweden | |  |
| HE_NC14-2 | Non-atrophic gastritis | | | | | | | | | Non Atrophic Gastritis | | | | | | Sweden | |  |
| HE_NC18-1 | Antrum Met, Corpus Atr + Met | | | | | | | | | Progressive towards Cancer | | | | | | Sweden | |  |
| HE_NC18-2 | Antrum Met, Corpus Atr + Met | | | | | | | | | Progressive towards Cancer | | | | | | Sweden | |  |
| HE_NC18-4 | Antrum Atr + Met, Corpus NAG | | | | | | | | | Progressive towards Cancer | | | | | | Sweden | |  |
| HE_NC13-5 | Antrum Met, Corpus NAG | | | | | | | | | Progressive towards Cancer | | | | | | Sweden | |  |
| HE_NC19-3 | Non-atrophic gastritis | | | | | | | | | Non Atrophic Gastritis | | | | | | Sweden | |  |
| HE_NC19-5 | Non-atrophic gastritis | | | | | | | | | Non Atrophic Gastritis | | | | | | Sweden | |  |
| HE_NC20-5 | Non-atrophic gastritis | | | | | | | | | Non Atrophic Gastritis | | | | | | Sweden | |  |
| HE_NC1-2 | Non-atrophic gastritis | | | | | | | | | Non Atrophic Gastritis | | | | | | Sweden | |  |
| HE_NC23-2a | Non-atrophic gastritis | | | | | | | | | Non Atrophic Gastritis | | | | | | Sweden | |  |
| HE_NC24-6 | Antrum NAG, Corpus Atr | | | | | | | | | Progressive towards Cancer | | | | | | Sweden | |  |
| HE_NC26-4 | Non-atrophic gastritis | | | | | | | | | Non Atrophic Gastritis | | | | | | Sweden | |  |
| HE_NC27-4 | Antrum NAG, Corpus Atr + Met | | | | | | | | | Progressive towards Cancer | | | | | | Sweden | |  |
| HE_NC29-2 | Antrum and Corpus Atr + Met | | | | | | | | | Progressive towards Cancer | | | | | | Sweden | |  |
| HE_NC30-2 | Non-atrophic gastritis | | | | | | | | | Non Atrophic Gastritis | | | | | | Sweden | |  |
| HE_NC30-3 | Non-atrophic gastritis | | | | | | | | | Non Atrophic Gastritis | | | | | | Sweden | |  |
| HE_NC32-4 | Antrum Met, Corpus NAG | | | | | | | | | Progressive towards Cancer | | | | | | Sweden | |  |
| HE_NC32-5 | Antrum Met, Corpus NAG | | | | | | | | | Progressive towards Cancer | | | | | | Sweden | |  |
| HE_NC5-3 | Non-atrophic gastritis | | | | | | | | | Non Atrophic Gastritis | | | | | | Sweden | |  |
| HE_NC36-3 | Non-atrophic gastritis | | | | | | | | | Non Atrophic Gastritis | | | | | | Sweden | |  |
| HE_NC38-2 | Antrum and Corpus Atr + Met | | | | | | | | | Progressive towards Cancer | | | | | | Sweden | |  |
| HE_NC38-4 | Antrum Met, Corpus NAG | | | | | | | | | Progressive towards Cancer | | | | | | Sweden | |  |
| HE_NC38-5 | Antrum and Corpus Atr + Met | | | | | | | | | Progressive towards Cancer | | | | | | Sweden | |  |
| HE_NC39-3 | Non Atrophic Gastritis | | | | | | | | | Non Atrophic Gastritis | | | | | | Sweden | |  |
| HE_NC47-5 | Antrum Met, Corpus NAG | | | | | | | | | Progressive towards Cancer | | | | | | Sweden | |  |
| HE_NC55-1 | Non-atrophic gastritis | | | | | | | | | Non Atrophic Gastritis | | | | | | Sweden | |  |
| HE_NC55-2 | Antrum Atr + Met, Corpus Atr | | | | | | | | | Progressive towards Cancer | | | | | | Sweden | |  |
| HE_NC55-5 | Non-atrophic gastritis | | | | | | | | | Non Atrophic Gastritis | | | | | | Sweden | |  |
| **Table S2, Isolate details for the hpEurope GWAS dataset. (page 4)** | | | | | | | | | | | | | | | | | |  |
| **Isolate** | **Host Pathology** | | | | | | | | | **GWAS group** | | | | | | **Isolation Country** | |  |
| HE_NC60-1 | Non-atrophic gastritis | | | | | | | | | Non Atrophic Gastritis | | | | | | Sweden | |  |
| HE_NC60-3 | Non-atrophic gastritis | | | | | | | | | Non Atrophic Gastritis | | | | | | Sweden | |  |
| HE_NC61-4 | Antrum Atr + Met, Corpus NAG | | | | | | | | | Progressive towards Cancer | | | | | | Sweden | |  |
| HE_NC89-4 | Non-atrophic gastritis | | | | | | | | | Non Atrophic Gastritis | | | | | | Sweden | |  |
| HE_NC9-1 | Non-atrophic gastritis | | | | | | | | | Non Atrophic Gastritis | | | | | | Sweden | |  |
| HE_NC11-1 | Antrum and Corpus Atrophy | | | | | | | | | Progressive towards Cancer | | | | | | Sweden | |  |
| 2012-26 | Metaplasia | | | | | | | | | Progressive towards Cancer | | | | | | Mexico | |  |
| 22025 | Gastritis | | | | | | | | | Non Atrophic Gastritis | | | | | | Colombia | |  |
| ms1055 | gastric cancer | | | | | | | | | Gastric Cancer | | | | | | Mexico | |  |
| 22402 | gastric cancer | | | | | | | | | Gastric Cancer | | | | | | Colombia | |  |
| 22087 | Gastritis | | | | | | | | | Non Atrophic Gastritis | | | | | | Colombia | |  |
| 26084 | gastric cancer | | | | | | | | | Gastric Cancer | | | | | | Colombia | |  |
| 2004-20 | Metaplasia | | | | | | | | | Progressive towards Cancer | | | | | | Mexico | |  |
| 2005-98 | Gastritis | | | | | | | | | Non Atrophic Gastritis | | | | | | Mexico | |  |
| ms203 | Gastritis | | | | | | | | | Non Atrophic Gastritis | | | | | | Mexico | |  |
| 26093 | gastric cancer | | | | | | | | | Gastric Cancer | | | | | | Colombia | |  |
| ms1078 | gastric cancer | | | | | | | | | Gastric Cancer | | | | | | Mexico | |  |
| 2006-52 | gastric cancer | | | | | | | | | Gastric Cancer | | | | | | Mexico | |  |
| 2006-407 | Gastritis | | | | | | | | | Non Atrophic Gastritis | | | | | | Mexico | |  |
| 22346 | Metaplasia | | | | | | | | | Progressive towards Cancer | | | | | | Colombia | |  |
| ms15 | Gastritis | | | | | | | | | Non Atrophic Gastritis | | | | | | Mexico | |  |
| 22337 | Atrophic Gastritis | | | | | | | | | Progressive towards Cancer | | | | | | Colombia | |  |
| ms23 | Gastritis | | | | | | | | | Non Atrophic Gastritis | | | | | | Mexico | |  |
| ms2 | Gastritis | | | | | | | | | Non Atrophic Gastritis | | | | | | Mexico | |  |
| 22341 | Metaplasia | | | | | | | | | Progressive towards Cancer | | | | | | Colombia | |  |
| 2006-56 | Metaplasia | | | | | | | | | Progressive towards Cancer | | | | | | Mexico | |  |
| 22023 | Gastritis | | | | | | | | | Non Atrophic Gastritis | | | | | | Colombia | |  |
| 22327 | Atrophic Gastritis | | | | | | | | | Progressive towards Cancer | | | | | | Colombia | |  |
| ms931 | gastric cancer | | | | | | | | | Gastric Cancer | | | | | | Mexico | |  |
| 2005-100 | Gastritis | | | | | | | | | Non Atrophic Gastritis | | | | | | Mexico | |  |
| ms1080 | gastric cancer | | | | | | | | | Gastric Cancer | | | | | | Mexico | |  |
| ms13 | Gastritis | | | | | | | | | Non Atrophic Gastritis | | | | | | Mexico | |  |
| 26100 | gastric cancer | | | | | | | | | Gastric Cancer | | | | | | Colombia | |  |
| 22046 | Metaplasia | | | | | | | | | Progressive towards Cancer | | | | | | Colombia | |  |
| **Table S2, Isolate details for the hpEurope GWAS dataset. (page 5)** | | | | | | | | | | | | | | | | | |  |
| **Isolate** | **Host Pathology** | | | | | | | | | **GWAS group** | | | | | | **Isolation Country** | |  |
| 2006-479 | Gastritis | | | | | | | | | Non Atrophic Gastritis | | | | | | Mexico | |  |
| 22389 | Gastritis | | | | | | | | | Non Atrophic Gastritis | | | | | | Colombia | |  |
| 22013 | Metaplasia | | | | | | | | | Progressive towards Cancer | | | | | | Colombia | |  |
| 22362 | Atrophic Gastritis | | | | | | | | | Progressive towards Cancer | | | | | | Colombia | |  |
| 2005-126 | Metaplasia | | | | | | | | | Progressive towards Cancer | | | | | | Mexico | |  |
| 2003-103 | Metaplasia | | | | | | | | | Progressive towards Cancer | | | | | | Mexico | |  |
| 22367 | Gastritis | | | | | | | | | Non Atrophic Gastritis | | | | | | Colombia | |  |
| 22021 | Metaplasia | | | | | | | | | Progressive towards Cancer | | | | | | Colombia | |  |
| 2006-480 | Metaplasia | | | | | | | | | Progressive towards Cancer | | | | | | Mexico | |  |
| 22385 | Gastritis | | | | | | | | | Non Atrophic Gastritis | | | | | | Colombia | |  |
| 2006-4 | Gastritis | | | | | | | | | Non Atrophic Gastritis | | | | | | Mexico | |  |
| 22370 | Gastritis | | | | | | | | | Non Atrophic Gastritis | | | | | | Colombia | |  |
| 22311 | Atrophic Gastritis | | | | | | | | | Progressive towards Cancer | | | | | | Colombia | |  |
| 22390 | Metaplasia | | | | | | | | | Progressive towards Cancer | | | | | | Colombia | |  |
| 22339 | Atrophic Gastritis | | | | | | | | | Progressive towards Cancer | | | | | | Colombia | |  |
| 22312 | Atrophic Gastritis | | | | | | | | | Progressive towards Cancer | | | | | | Colombia | |  |
| 22322 | Gastritis | | | | | | | | | Non Atrophic Gastritis | | | | | | Colombia | |  |
| 22331 | Metaplasia | | | | | | | | | Progressive towards Cancer | | | | | | Colombia | |  |
| 26024 | Atrophic Gastritis | | | | | | | | | Progressive towards Cancer | | | | | | Colombia | |  |
| 22368 | Metaplasia | | | | | | | | | Progressive towards Cancer | | | | | | Colombia | |  |
| 22360 | Metaplasia | | | | | | | | | Progressive towards Cancer | | | | | | Colombia | |  |
| 22378 | Gastritis | | | | | | | | | Non Atrophic Gastritis | | | | | | Colombia | |  |
| 22019 | Atrophic Gastritis | | | | | | | | | Progressive towards Cancer | | | | | | Colombia | |  |
| 22020 | Atrophic Gastritis | | | | | | | | | Progressive towards Cancer | | | | | | Colombia | |  |
| 22315 | Metaplasia | | | | | | | | | Progressive towards Cancer | | | | | | Colombia | |  |
| 22335 | Atrophic Gastritis | | | | | | | | | Progressive towards Cancer | | | | | | Colombia | |  |
| ms176 | Gastritis | | | | | | | | | Non Atrophic Gastritis | | | | | | Mexico | |  |
| 22393 | Atrophic Gastritis | | | | | | | | | Progressive towards Cancer | | | | | | Colombia | |  |
| 22093 | gastric cancer | | | | | | | | | Gastric Cancer | | | | | | Colombia | |  |
| 22095 | Atrophic Gastritis | | | | | | | | | Progressive towards Cancer | | | | | | Colombia | |  |
| 22347 | Metaplasia | | | | | | | | | Progressive towards Cancer | | | | | | Colombia | |  |
| 2011-41 | Gastritis | | | | | | | | | Non Atrophic Gastritis | | | | | | Mexico | |  |
| 22388 | Metaplasia | | | | | | | | | Progressive towards Cancer | | | | | | Colombia | |  |
| 22351 | Metaplasia | | | | | | | | | Progressive towards Cancer | | | | | | Colombia | |  |
| **Table S2, Isolate details for the hpEurope GWAS dataset. (page 6)** | | | | | | | | | | | | | | | | | |  |
| **Isolate** | **Host Pathology** | | | | | | | | | **GWAS group** | | | | | | **Isolation Country** | |  |
| 22384 | Gastritis | | | | | | | | | Non Atrophic Gastritis | | | | | | Colombia | |  |
| 24008 | Atrophic Gastritis | | | | | | | | | Progressive towards Cancer | | | | | | Colombia | |  |
| ms965 | gastric cancer | | | | | | | | | Gastric Cancer | | | | | | Mexico | |  |
| * Hp population according to fineSTRUCTURE analysis in Figure S1 | | | | | | | | | | | | | | | |  | |  |
| NAG = Non-antrophic gastritis, Atr = Atrophy, Met = Metaplasia, IT = Intestinal type, DT = Diffuse type | | | | | | | | | | | | | | | | | |  |

**Table S3, List of the 32 genes highlighted in at least one of the GWAS experiments**

| **Gene name** | **Minimum p-value** | **Prokka Annotation** |
| --- | --- | --- |
| HP1055 | 1,40E-09 | Helicobacter outer membrane protein |
| HP0797 | 2,24E-08 | Neuraminyllactose-binding hemagglutinin precursor (NLBH) |
| HP1243 | 3,99E-08 | Helicobacter outer membrane protein |
| HP0555 | 5,58E-08 | TrbL/VirB6 plasmid conjugal transfer protein |
| HP0747 | 1,69E-07 | tRNA (guanine-N(7)-)-methyltransferase |
| HP0709 | 2,13E-07 | Adenosyl-chloride synthase |
| HP1004 | 2,73E-07 | Type III restriction enzyme, res subunit |
| HP0532 | 3,62E-07 | CAG pathogenicity island protein 12 precursor |
| HP0468 | 4,59E-07 | hypothetical protein |
| HP0531 | 5,40E-07 | hypothetical protein |
| HP0541 | 6,60E-07 | hypothetical protein |
| HP0906 | 7,08E-07 | Flagellar hook-length control protein FliK |
| HP1331 | 1,06E-06 | Inner membrane protein YgaZ |
| HP0068 | 1,42E-06 | Urease accessory protein UreG |
| HP1421 | 2,26E-06 | Type IV secretion system protein PtlH |
| HP1046 | 2,27E-06 | Ribosome maturation factor RimP |
| HP0540 | 2,33E-06 | hypothetical protein |
| HP0527 | 2,34E-06 | Type IV secretion system protein virB10 |
| HP0524 | 2,53E-06 | Conjugal transfer protein TraG |
| HP1149 | 3,46E-06 | Ribosome maturation factor RimM |
| HP0102 | 4,49E-06 | PGL/p-HBAD biosynthesis glycosyltransferase/MT3031 |
| HP0528 | 4,54E-06 | Conjugal transfer protein |
| HP0615 | 4,94E-06 | Heme transporter BhuA precursor |
| HP0290 | 5,88E-06 | Diaminopimelate decarboxylase |
| HP1572 | 5,90E-06 | Membrane-bound lytic murein transglycosylase D precursor |
| HP0269 | 5,90E-06 | (Dimethylallyl)adenosine tRNA methylthiotransferase MiaB |
| HP0936 | 7,24E-06 | Proline/betaine transporter |
| HP1177 | 7,48E-06 | Helicobacter outer membrane protein |
| HP0544 | 7,92E-06 | Type IV secretion system protein virB4 |
| HP1184 | 8,65E-06 | Multidrug export protein MepA |
| HP0569 | 8,86E-06 | Ribosome-binding ATPase YchF |
| HP1460 | 9,73E-06 | DNA polymerase III subunit alpha |
